# A Predictive Model of the Oxygen and Heme Regulatory Network in Yeast

**DOI:** 10.1371/journal.pcbi.1000224

**Published:** 2008-11-14

**Authors:** Anshul Kundaje, Xiantong Xin, Changgui Lan, Steve Lianoglou, Mei Zhou, Li Zhang, Christina Leslie

**Affiliations:** 1 Department of Computer Science, Columbia University, New York, New York, United States of America; 2 Department of Molecular and Cell Biology, University of Texas at Dallas, Richardson, Texas, United States of America; 3 Department of Physiology, Biophysics, and Systems Biology, Weill Medical College of Cornell University, New York, New York, United States of America; 4 Computational Biology Program, Memorial Sloan-Kettering Cancer Center, New York, New York, United States of America; University of Washington, United States of America

## Abstract

Deciphering gene regulatory mechanisms through the analysis of high-throughput expression data is a challenging computational problem. Previous computational studies have used large expression datasets in order to resolve fine patterns of coexpression, producing clusters or modules of potentially coregulated genes. These methods typically examine promoter sequence information, such as DNA motifs or transcription factor occupancy data, in a separate step after clustering. We needed an alternative and more integrative approach to study the oxygen regulatory network in *Saccharomyces cerevisiae* using a small dataset of perturbation experiments. Mechanisms of oxygen sensing and regulation underlie many physiological and pathological processes, and only a handful of oxygen regulators have been identified in previous studies. We used a new machine learning algorithm called MEDUSA to uncover detailed information about the oxygen regulatory network using genome-wide expression changes in response to perturbations in the levels of oxygen, heme, Hap1, and Co^2+^. MEDUSA integrates mRNA expression, promoter sequence, and ChIP-chip occupancy data to learn a model that accurately predicts the differential expression of target genes in held-out data. We used a novel margin-based score to extract significant condition-specific regulators and assemble a global map of the oxygen sensing and regulatory network. This network includes both known oxygen and heme regulators, such as Hap1, Mga2, Hap4, and Upc2, as well as many new candidate regulators. MEDUSA also identified many DNA motifs that are consistent with previous experimentally identified transcription factor binding sites. Because MEDUSA's regulatory program associates regulators to target genes through their promoter sequences, we directly tested the predicted regulators for *OLE1*, a gene specifically induced under hypoxia, by experimental analysis of the activity of its promoter. In each case, deletion of the candidate regulator resulted in the predicted effect on promoter activity, confirming that several novel regulators identified by MEDUSA are indeed involved in oxygen regulation. MEDUSA can reveal important information from a small dataset and generate testable hypotheses for further experimental analysis. Supplemental data are included.

## Author Summary

The cell uses complex regulatory networks to modulate the expression of genes in response to changes in cellular and environmental conditions. The transcript level of a gene is directly affected by the binding of transcriptional regulators to DNA motifs in its promoter sequence. Therefore, both expression levels of transcription factors and other regulatory proteins as well as sequence information in the promoters contribute to transcriptional gene regulation. In this study, we describe a new computational strategy for learning gene regulatory programs from gene expression data based on the MEDUSA algorithm. We learn a model that predicts differential expression of target genes from the expression levels of regulators, the presence of DNA motifs in promoter sequences, and binding data for transcription factors. Unlike many previous approaches, we do not assume that genes are regulated in clusters, and we learn DNA motifs de novo from promoter sequences as an integrated part of our algorithm. We use MEDUSA to produce a global map of the yeast oxygen and heme regulatory network. To demonstrate that MEDUSA can reveal detailed information about regulatory mechanisms, we perform biochemical experiments to confirm the predicted regulators for an important hypoxia gene.

## Introduction

Oxygen is critical for the survival and development of virtually all living organisms. As such, living organisms ranging from yeast to humans have developed sophisticated mechanisms to respond to changes of oxygen level in the environment [1]. Several microarray gene expression studies have been performed in the yeast model eukaryote *Saccharomyces cerevisiae* to understand oxygen sensing and regulation at a genome-wide level [2]–[6]. However, most of these studies mainly identified genes responding to low levels of oxygen [2]–[6] or determined the DNA-binding sites for several known oxygen regulators, such as Rox1 [3]. Recently, there has also been a cluster analysis of expression profiles under hypoxia and reoxygenation in glucose versus galactose media [6],[7], where the authors looked for enrichment of functional annotations and known transcription factor binding sites within gene clusters and also applied existing motif discovery algorithms to the clusters. These previous microarray studies have provided further evidence of the role of known regulators such as Hap1, Rox1, and Upc2, but they have had limited success in identifying novel components of the oxygen and heme regulatory network.

In this study, we apply an integrative computational approach to analyze genome-wide changes in expression in response to perturbations of the oxygen regulatory network. Our approach is based on a new machine learning algorithm called MEDUSA that combines information from promoter sequences and gene expression profiling to learn a quantitative and statistically robust global model for the oxygen regulatory system. (A mathematical description of MEDUSA has appeared as an extended abstract in a conference proceedings [8]). Numerous computational approaches for inferring gene regulatory networks from gene expression data have been developed to date [9]–[20]. We were motivated by two recent computational approaches in particular: one algorithm aimed at predicting a gene's cluster membership based on the motifs in its promoter [21], and another for partitioning genes into regulatory modules, i.e., clusters of genes with shared regulatory logic [22]. Both these previous methods depend on clustering genes and describing regulatory logic at the level of clusters. However, genes with similar expression patterns are not necessarily regulated by the same regulators and mechanisms. Thus, in our work, we do not assume that clusters reflect the full complexity and condition-specific nature of gene regulation. Indeed, given that virtually all yeast RNA polymerase II promoters are distinct, it remains to be demonstrated that any two promoters are regulated identically by the same regulators across all possible experimental conditions.

In contrast to these previous approaches, the MEDUSA algorithm aims to predict the condition-specific differential expression of individual genes, not clusters of genes, by using a single global model. It integrates promoter sequence, promoter occupancy data from ChIP-chip experiments, and the expression levels of potential regulators, including those that do not bind to DNA, to learn a regulatory program controlling target genes. Notably, MEDUSA identifies motifs directly from promoter sequences; no prior knowledge of any DNA-binding motifs is used. MEDUSA trains on differentially expressed target genes from multiple experiments to discover both the motifs in promoters and the condition-specific regulators that together define a global regulatory control program. This regulatory program predicts whether a gene will be up- or downregulated, given its promoter sequence and the condition-specific expression level of the regulators. MEDUSA uses a modern statistical learning technique called boosting [23] to avoid over-fitting as it searches through the large space of possible regulators and sequence motifs [24]. As a result, it achieved high prediction accuracy in cross-validation results using held out gene-experiment examples for the oxygen regulation dataset, where we compare up/down prediction to experimentally measured differential expression, despite the fact that the number of gene expression experiments (6 conditions with 3 replicates) was much smaller than in previous computational approaches for learning regulatory networks. We then used a novel margin-based score to extract the condition-specific regulators and putative DNA binding site motifs that are most significant for predicting the expression of particular sets of target genes. We summarized this information with a global map of the oxygen regulatory network, which includes both known and novel regulators. Since MEDUSA associates regulators to target genes via motifs in the promoter sequence, we directly tested the predicted regulators for the *OLE1* gene by experimental analysis of its promoter activity under deletion of each of these regulators. In each case, the change in *OLE1*'s promoter activity under hypoxia was consistent with MEDUSA's predictions. These results confirm that several novel regulators are indeed involved in oxygen regulation. Finally, we performed a comprehensive comparison of the motif discovery results of MEDUSA with a conventional cluster-first motif discovery algorithm, and we found that MEDUSA identified many DNA binding site motifs that are relevant to hypoxia and missed by the traditional approach.

## Results

### Perturbations of the Oxygen Regulatory Network Reveal Diverse Expression Signatures

We used microarray gene expression profiling data from triplicate RNA samples, prepared from cells grown under eight different experimental conditions (see Methods) to observe perturbations of the oxygen sensing and regulatory network and study its behavior. We identified several classes of genes: Hap1-dependent or -independent oxygen-regulated, heme-regulated, and Co^2+^-inducible genes (see Figures S6 and S7). These results were consistent with previous studies identifying differentially expressed target genes. For example, we identified previously known oxygen- and heme-induced genes, such as *COXs*, *CYC7* and *CYT1* [25]–[29]; and previously known hypoxia-induced genes, such as *ANB1*, *MGA2*, *OLE1*, *PAU2-5*, *PAU7*, *DAN1-4* and *HSPs* genes [30]–[39].

**Figure 1:**
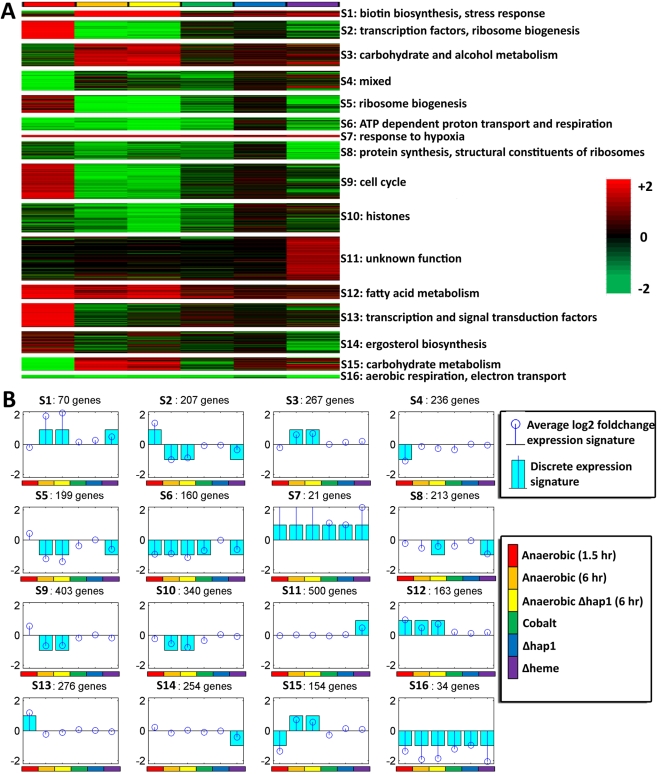
Expression signatures identified by perturbation of the oxygen regulatory network. (A) Heat maps showing real-valued expression profiles of genes that are members of the 16 signatures identified. The expression values are in log_2_. The rows represent genes and the columns represent the 6 experimental conditions. Bright red indicates strong upregulation, bright green indicates strong downregulation, and black indicates no change in expression. Each signature is labeled with statistically significant functional annotations. (B) Each block displays the average real-valued expression (stem plot in dark blue) and discrete expression profile (bar plot in light blue) for each signature over the 6 experimental conditions. The real-valued expression values are in log_2_.

Prior to performing more integrative computational analysis, we also examined the broad patterns of gene expression in our dataset. We identified 16 distinct discretized co-expression signatures (see Methods) to which we assigned the differentially expressed genes, including 5 pairs of antagonistic signatures whose patterns of expression are nearly the same but opposite in direction (Figure 1). For example, we found a pair of expression signatures consisting of genes that are exclusively induced by heme deletion (signature 11) or exclusively suppressed in this condition (signature 14). Signature 14 contains the ergosterol biosynthesis genes, which are known to be heme regulated, while most of the genes in signature 11 (354 out of 500) are functionally uncharacterized and may include novel heme-regulated genes. While most of these expression signatures contain several hundred genes, a few sets are smaller and functionally more coherent. In particular, signature 16 consists of 34 Hap1-dependent genes that are strongly suppressed in all conditions including the *Δhap1* experiment. These genes include the *COX* and *QCR* genes and are involved in aerobic respiratory processes, electron transport, and heme-dependent oxidoreducatase activity. In most cases, the expression signatures could be assigned significant functional terms, though in general only a smaller subset of the genes in a signature belong to the enriched category (see Text S1 for details).

### MEDUSA Deciphers Regulatory Networks without Clustering Genes

While these signatures consist of genes that are coexpressed, for the most part they are large and functionally heterogeneous gene sets and do not represent true functional regulons. In general, small expression datasets may not contain enough resolution to decipher fine context-specific coexpression patterns, and analysis of coexpressed gene sets can provide only limited insight into regulation in this case. Applying typical motif discovery algorithms to broadly coexpressed gene sets also leads to problems (see results below), since the gene “clusters” are in fact likely to be mixtures of differently regulated gene sets. However, the strength of our dataset is that it measures key perturbations related to oxygen and heme regulation; it should contain rich information about the commonalities and differences in regulation of the genes that respond to these perturbations. Therefore, instead of using coexpressed gene sets as independent inputs for subsequent computational analysis, we used MEDUSA to learn a gene regulatory program from the entirety of the perturbation data.

MEDUSA does not depend only on the correlation of the mRNA level of regulators and targets to infer a regulatory relationship. Instead, the algorithm requires that regulators control their targets through the presence of shared sequence motifs in the promoters of target genes, and it learns these motifs as it builds a regulatory program. MEDUSA integrates a number of ideas that have been used previously in computational modeling of regulatory programs but also implements a conceptually different approach. For example, like the module networks algorithm of Segal et al. [22], we used the expression levels of a known set of transcriptional regulators, which include both transcription factors and signaling transducers, to learn a context-specific model of regulation. However, while the module networks approach uses only expression data, MEDUSA also incorporates promoter sequence data to learn DNA motifs as part of the regulatory program. As in recent work of Beer and Tavazoie [21], we wish to predict expression from promoter sequences without reference to a target gene's identity. However, rather than predicting the cluster membership of genes, our model predicts up and down regulation of individual target genes, based on promoter sequence and regulator expression, across multiple experimental conditions.

MEDUSA also differs from these previous studies and most other work by implementing a number of key algorithmic features: (1) it integrates promoter sequence, expression and ChIP-chip data to predict a global regulatory program; (2) it avoids over-fitting when training in a high dimensional feature space by use of a machine learning technique called boosting; (3) it learns functional contributions of both regulators and motifs; (4) it learns binding site motifs directly from sequence without seeding the algorithm with known motifs; (5) it automatically learns the threshold for deciding the presence of motifs. We note that in our previous work [40],[41], we used boosting to learn regulatory models based on a library of known motifs, while MEDUSA learns motifs de novo. In an earlier work, Segal et al. [42] used a probabilistic relational model framework to learn transcriptional modules, i.e., gene sets whose shared expression patterns are supported by the shared motifs in their promoter sequences. Unlike our approach, this algorithm relies on assigning genes to static clusters and is seeded with database motifs, though motifs are re-estimated over training iterations. More recently, for the special case of time series expression data, Ernst et al. [43] presented a probabilistic approach that learns a temporally-organized hierarchical clustering of genes, where bifurcations of genes that go up or down at specific time points are explained by shared motifs or ChIP chip occupancy data. This algorithm uses static occupancy and database motif data rather than learning motifs at the same time as the regulation model.

**Figure 2:**
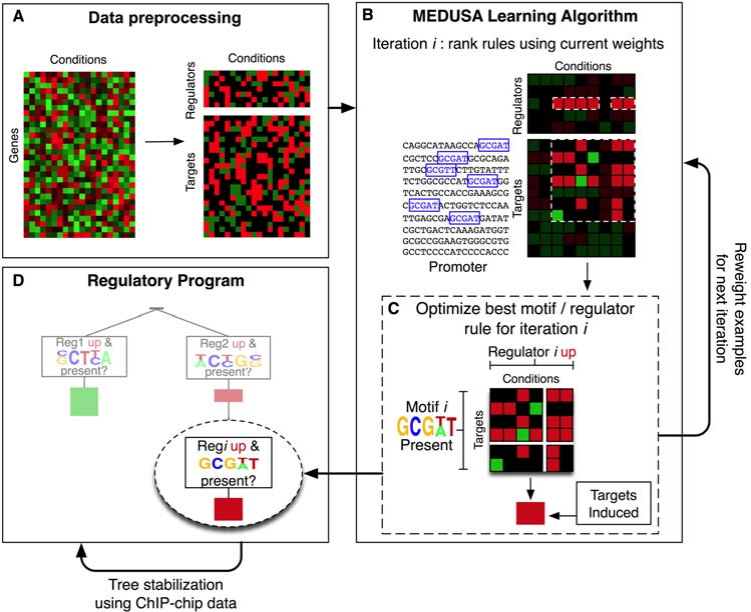
A schematic flow chart showing the algorithmic steps for learning the oxygen regulatory program with MEDUSA. (A) The mRNA expression data is discretized into three states, up (over-expressed), down (under-expressed), and baseline (not significantly differentially expressed), and genes are partitioned into potential regulators (transcription factors and signal transducers) and targets. The regulators are also included in the list of target genes so that their transcriptional regulation can be modeled. (B) The MEDUSA learning algorithm is presented with the promoter sequences of target genes, the discretized expression profiles of the regulators across multiple conditions, and the differentially expressed (up and down) target gene examples from these experiments. Baseline examples are not used to train MEDUSA. In the first stage of training, MEDUSA considers rules based on promoter sequence data and regulator expression states. MEDUSA uses a boosting strategy to avoid overfitting over many rounds of the algorithm. At each iteration *i*, a motif/regulator rule is chosen based on the current weights on the training examples; this rule predict that targets whose promoters contain the motif will go up (or down) in experiments where the regulator is over- (or under-) expressed. Before the next iteration, the examples are reweighted to emphasize the ones that are difficult to predict. (C) To learn the sequence motif, the algorithm agglomerates predictive *k*-mer sequences to produce candidate PSSMs, and it optimizes both the choice of PSSM and the probabilistic threshold used to determine where the hits of the motif occur. (D) At the end of each round of training, motif /regulator rules are placed into an alternating decision tree, building a global regulatory program. This regulatory program can be used to predict target gene up/down regulation for gene-experiment examples that were not seen in training. In order to produce a more stable decision tree, we perform a second pass of the tree-learning algorithm using a stabilized variant of boosting that gives more consistent models over different subsets of the training data. At this stage, both the motifs learned previously by MEDUSA and TF occupancies from ChIP-chip experiments are used as sequence features for the final regulatory program.

**Figure 3:**
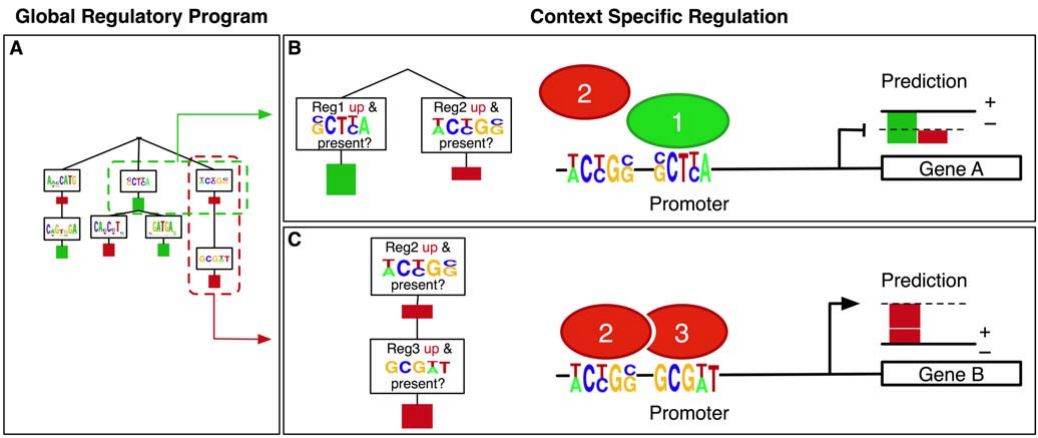
Simplified example showing how the regulatory program learned by MEDUSA predicts context-specific up/down gene expression. MEDUSA learns a global regulatory program described by an alternating decision tree. A simple regulatory program is shown in part A of the figure, along with the prediction it makes in two contexts, indicated as context B (top right) and context C (bottom right). The interaction between a regulator and a motif and the effect on targets is described by a decision node, which contains a logical condition to be tested, e.g., “Is regulator *i* up in the experiment and is motif *i* present in the promoter?”, and by the contribution that this motif/regulator pair makes to the up/down prediction of target gene expression if the logical condition is true, which is indicated by a colored bar. Contributions to upregulation of targets are shown in red and downregulation of targets in green. Combinatorial regulation is encoded by the tree structure: we obtain a prediction score for the up/down regulation of a target gene in a given experimental condition by starting at the root and recursively working downwards in the tree, seeing which prediction nodes are reachable by answering “yes” to logical conditions and summing all score contributions for the nodes visited. (Context B) In the first context, both Reg 2, a transcriptional activator, and Reg 1, a repressor, are expressed, and the promoter of gene *A* contains the motifs associated by the regulatory program to both these regulators. The regulatory program computes the prediction score by summing the larger contribution of the repressor (green bar) with the smaller contribution of the activator (red bar) to obtain a negative prediction score (indicated by the dashed line on the far right), i.e., gene *A* is predicted to be downregulated. (Context C) In the second context, both the activator Reg 2 and a co-factor, Reg 3, are expressed and can bind to the promoter of gene *B* based on the presence of the associated motifs in the regulatory program. The logic of the tree requires that the condition involving Reg 2 must hold before the contribution of the node containing Reg 3, at the next level of the tree, can be counted. Here, both conditions hold, and the regulatory program adds two positive contributions to obtain a confident prediction that gene *B* will be upregulated.

Figure 2 illustrates the major steps and data used in the MEDUSA learning algorithm. In preprocessing, mRNA expression data is discretized by binning expression values into three states (up, down, and baseline) and partitioning genes into regulators and targets (Figure 2A). In the first stage of training (Figure 2B and 2C), MEDUSA uses the promoter sequences of target genes and the mRNA levels of regulators as inputs to learn a prediction function for the differential expression of targets. MEDUSA uses boosting to iteratively discover motifs whose presence in the promoters of target genes, together with the mRNA levels of regulators across experimental conditions, helps to predict the differential expression of the targets in those conditions. It builds a global regulatory program based on these motifs and regulators (Figure 2D). In order to produce a regulatory program that is more consistent under variations of the training data, a second pass of the regulatory program building algorithm is performed using a stabilized variant of boosting (see Methods). This second pass integrates the motifs discovered in the first training stage, promoter occupancy data from ChIP-chip analysis, and expression data of regulators and targets, to build a final regulatory program that predicts the up or down regulation of target genes. The regulatory program asks questions such as, “Is the mRNA level of regulator *ρ* up (or down) in the experiment, and is the motif *μ* present in the upstream region of the gene (or is a transcriptional regulator bound to the promoter, when ChIP-chip data are available)?” The control logic of the regulatory program is described by an alternating decision tree (Figure 2D and Figure 3), which encodes how the overall up or down prediction score for a target gene in a given experimental condition results from the contribution and interaction of multiple regulators and motifs.

Figure 3 presents a simplified example to illustrate how a regulatory program generated by MEDUSA computes a context-specific prediction score to predict the up/down regulation of target genes. The size of the score can be interpreted as our confidence in the up/down prediction. In context B (Figure 3, top right), the promoter of gene *A* contains a pair of sequence motifs associated by the regulatory program to a weak activator and a stronger repressor that are both expressed in the experimental condition. The regulatory program makes a moderately confident prediction that gene *A* will be downregulated (depicted by the dashed line on the extreme right), based on the sum of scores from the relevant pair of nodes in the tree. In context C (Figure 3, bottom right), the promoter of target gene *B* contains binding sites for the weak activator but also for a co-factor, placed in a node below the weak activator in the tree. Both the activator and the co-factor are expressed in the condition shown, and the regulatory program computes a confident up prediction for gene *B* in this condition. In this way, MEDUSA encapsulates a genome-wide and context-specific regulatory program, learned directly from promoter sequence and expression data and without the introduction of additional prior assumptions.

### MEDUSA Achieves High Prediction Accuracy in Cross-Validation

We first performed a statistical analysis to verify that MEDUSA could achieve good prediction performance on held-out data, i.e., correctly predict up or down expression for examples not included in the training data. In our cross-validation experiments, we used 90% of the gene-condition experiments as the training set to identify statistically significant regulators and motifs. Then, we used these regulators and motifs to predict the expression of the 10% held-out examples, as illustrated in Figure 3 (see also Text S1). We achieved 92% accuracy across 10-fold cross-validation experiments in the prediction of the up or down expression of held-out examples (Table 1). MEDUSA is trained to solve the binary prediction problem of discriminating between upregulated and downregulated examples. Such differentially expressed examples constitute about 12% of our dataset, and a total of 3482 genes were differentially expressed in at least one condition. However, by thresholding the positive and negative real-valued prediction scores, we can make three-class predictions of up, down, or baseline across all held-out examples, not just the differentially expressed ones. We found that MEDUSA's accuracy on the three-class prediction problem was still high; in particular, true baseline examples were most often predicted to be baseline, even though no baseline examples were seen during training (see Text S1, Figure S1, and Figure S2).

**Table 1:** Tenfold cross-validation accuracy for nearest-regulator method and MEDUSA, with and without grouping replicate examples within a fold.

Prediction Method	Tenfold c.v Accuracy (held-out examples)	Tenfold c.v Accuracy (replicates in same fold)
Nearest regulator	59.8%	31.0%
Majority vote of *k* = 10 nearest regulators	88.9%	55.0%
MEDUSA	92.0%	76.1%

We then investigated whether MEDUSA's high accuracy was simply an indication that the prediction task itself was easy, perhaps due to the presence of replicate experiments in the dataset; note, however, that one of the three replicates was performed on a different yeast strain (see Methods), so that the variation was higher than in technical replicates. We conducted a series of additional experiments (Table 1) to address this issue. We first compared MEDUSA's results to a simple correlation-based method, where we identified the regulator that best correlated with each target gene across the training examples (using a Hamming distance metric, see Methods) and used this regulator's expression state to predict the target's expression level on the test examples. This approach necessarily uses the target gene's identity rather than learning a single model that can be applied to all genes. We found that using a single “nearest regulator” for each target gave poor prediction results (similar or worse than random guessing, see Methods). Taking the majority vote over a set of 10 nearest regulators gave good test accuracy, but the regulators most frequently chosen as nearest regulators did not include any of the key known regulators; in fact, every regulator was chosen at least once, and key regulators like Hap1, Rox1, and Mga2 ranked low in terms of frequency. We then prepared a much more difficult cross-validation experiment, where all replicate examples of each gene in an experimental condition were grouped together in a fold. In this way, for any particular target gene, the training and test examples came from completely different conditions. In this setting, MEDUSA still achieved significant accuracy given that there were only 6 experimental conditions (76%), while the nearest regulator methods were no better than random guessing (Table 1). Finally, we ran MEDUSA using ChIP-chip occupancy features alone, without including promoter sequence data for motif discovery. We found that cross-validation accuracy deteriorated considerably (from 92% to 74%), indicating that motif discovery, or at least the use of sequence motifs, is crucial to MEDUSA's success (see Text S1). These experimental results strongly suggest that MEDUSA has learned regulatory information that generalizes to new data rather than over-fitting the training data.

Ideally, one would like to compare MEDUSA's cross-validation accuracy and predicted regulators to previous computational methods. Here, however, the relatively small size of our dataset precludes a reasonable comparison. Neither classical Bayesian network approaches [15] nor more recent module-oriented algorithms [21],[22] were designed to be trained on a small number of conditions, and indeed they have only been tested when very large expression datasets (>100 experiments) are available. Other motif discovery approaches such as REDUCE [44] use single microarray experiments as input but are designed to extract a small number of strong motifs that account for a statistically significant percentage of the within-experiment variance. Such methods are not optimized to accurately predict up/down expression, nor do they directly identify regulators, so it is difficult to set up a meaningful comparison. However, we do perform a comparison with standard cluster-based analysis and motif discovery below, where we show that both at a global level and when restricting attention to particular functional regulons, MEDUSA finds more detailed motif information (see below).

### MEDUSA Identifies a Network of Condition-Specific Regulators Mediating Global Gene Regulation

MEDUSA's high accuracy on held-out examples gives us confidence that the decision tree contains statistically significant and biologically relevant regulators and motifs. In order to identify the most significant regulators and motifs controlling specific sets of differentially expressed target genes under specific experimental conditions, we ranked these features using a novel margin-based score (see Methods). The score assesses how significantly an individual feature contributes to the confidence of predictions over the specific set of target genes and experiments.

**Figure 4:**
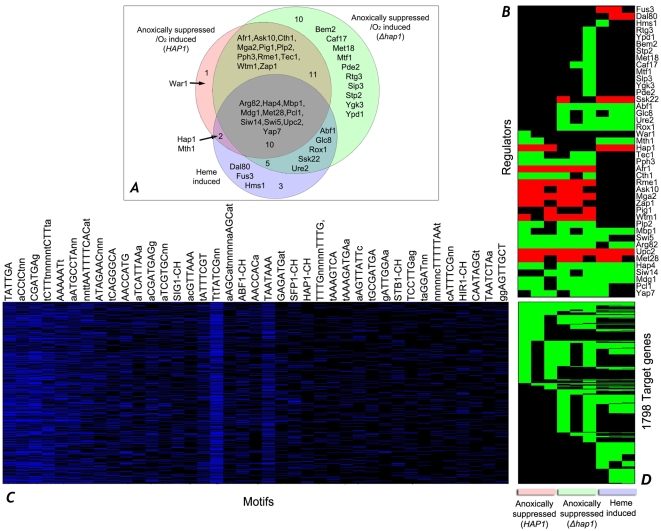
Heat maps showing predictive regulators, predictive motifs, and targets induced by oxygen and heme. (A) A Venn diagram illustrating the regulators involved in controlling hypoxically suppressed (oxygen-induced) genes in *HAP1* and *Δhap1* cells, and heme-induced genes. For each experiment, the statistically significant regulators associated with the set of downregulated target genes are determined by use of a margin-based score (see Methods). (B) Patterns of up (red), down (green), and baseline (black) expression levels for the statistically significant regulators controlling downregulated target genes across the three experimental conditions. (C) The top-ranked sequence features learned by MEDUSA, as determined by a margin-based score, and their hits across the set of target gene promoters. The PSSMs learned by MEDUSA are represented by their consensus patterns. ChIP-chip occupancy features also occur in the list of most significant features. For example, SIG1-CH refers to ChIP-chip occupancy by the transcription factor *SIG1* and appears as a highly-ranked promoter sequence feature. The presence or absence of a sequence feature in a gene's promoter is represented by blue or black blocks respectively. (D) Discretized gene expression levels for the full set of target genes represented in the Venn diagram (total of 1798 genes), given by combining the down-regulated target gene list from each of the three experimental conditions. Note that the expression patterns include only down and baseline expression levels across all three conditions.

We first used margin scoring to identify statistically significant regulators that may mediate the regulation of various target genes in the dataset. Figure 4 provides an example of the condition-specific regulators and motifs identified by MEDUSA. To clarify the roles of Hap1 and heme in oxygen regulation, we identified and compared the potential regulators (Figure 4A) and motifs (Figure 4B) that may mediate the regulation of hypoxically suppressed (oxygen-induced) genes in wild type *HAP1* or *Δhap1* cells, and heme-induced (heme deficiency-suppressed) genes. Note that intracellular heme levels are low under hypoxic growth conditions [45], so hypoxically suppressed (oxygen-induced) genes correlate with heme deficiency-suppressed (heme-induced) genes. The expression levels of identified regulators and target genes are indicated in Figure 4C and 4D, respectively.

MEDUSA analysis identified a number of known regulators whose predicted condition-specific role is consistent with previous knowledge of oxygen and heme regulation. For example, consistent with existing knowledge [33],[34],[46], Hap1 is important for the regulation of oxygen-induced genes in cells bearing the Hap1 expression plasmid and for heme induction of target genes (see Figure 4A and 4C). Likewise, Hap4 is important for heme induction and for the regulation of oxygen-induced genes in both cells bearing the Hap1 expression plasmid (*HAP1*) and the empty vector (*Δhap1*, Figure 4A and 4C). Rox1 appears to be important for the regulation of oxygen-induced genes only in *Δhap1* cells. This is not surprising because Rox1 expression is known to be under the control of Hap1 33,34. In cells bearing the Hap1 expression plasmid (*HAP1*), Hap1 would be the dominant regulator. Another notable case is Mga2, which has been shown to be important for oxygen regulation of certain genes, such as *OLE1* [31],[47]. Here we found that it is indeed important for oxygen induction of genes in both cells bearing the Hap1 expression plasmid (*HAP1*) and the empty vector, but it is not important for heme regulation, as expected.

**Figure 5:**
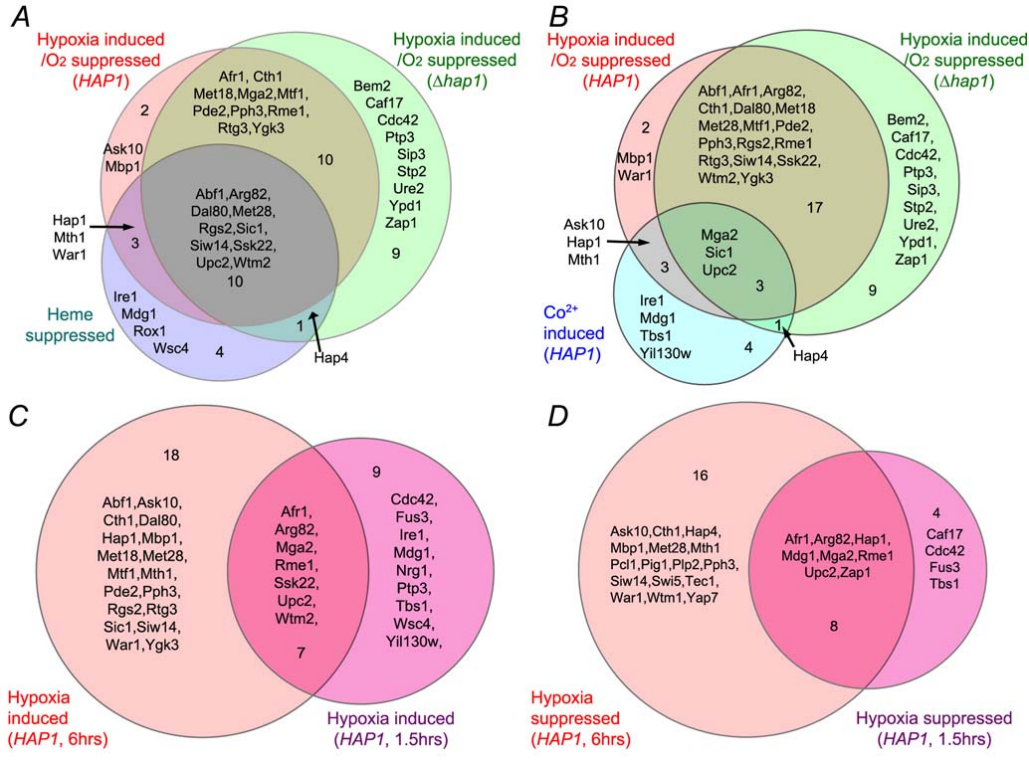
Venn diagrams showing the statistically significant, high ranking regulators mediating the regulation of oxygen-regulated, heme-regulated, and Co^2+^-inducible genes in *HAP1* and *Δhap1* cells. (A) A Venn diagram illustrating the regulators involved in controlling hypoxically induced (oxygen-suppressed) genes in *HAP1* and *Δhap1* cells, and heme-suppressed genes. (B) A Venn diagram illustrating the regulators involved in controlling hypoxically induced (oxygen-suppressed) genes in *HAP1 and Δhap1* cells, and Co^2+^-inducible genes. (C) A Venn diagram illustrating the regulators involved in controlling hypoxically induced (oxygen-suppressed) genes in *HAP1* cells at 1.5 or 6 hours after shifting to anaerobic growth conditions. (D) A Venn diagram illustrating the regulators involved in controlling hypoxically suppressed (oxygen-induced) genes in *HAP1* cells at 1.5 or 6 hours after shifting to anaerobic growth conditions.

We also identified and compared statistically significant regulators that may mediate the regulation of hypoxically induced genes in cells bearing the Hap1 expression plasmid (*HAP1*) and the empty vector (*Δhap1*) and those that mediate heme deficiency-induced (heme-suppressed) genes (Figure 5A and Figure S3). Likewise, we identified and compared regulators that may mediate the regulation of Co^2+^-inducible genes with those mediating the regulation of hypoxically induced genes (Figure 5B and Figure S4). The comparison of these regulators mediating oxygen regulation, heme regulation, and Co^2+^-inducible regulation provides several important insights into the regulatory network mediating oxygen sensing and regulation. First, more than half of the MEDUSA-identified regulators mediating heme regulation may also be involved in mediating oxygen regulation both in *HAP1* cells (12 out of 20 regulators) and in *Δhap1* cells (15 out of 20 regulators) (Figure 4A). Many regulators predicted to be involved in heme suppression of target genes may also be involved in oxygen induction in wild type *HAP1* cells (13 out of 18) and in *Δhap1* cells (11 out of 18) (Figure 5A). These results are consistent with the previous idea that heme serves as a secondary messenger of oxygen and plays a major role in mediating oxygen regulation of target genes. Second, Hap1 plays a major role in oxygen regulation. In the absence of Hap1, the number of regulators mediating oxygen regulation may be significantly increased both for oxygen-induced genes (Figure 4A) and hypoxically induced genes (Figure 5A).

Third, relatively few regulators may be involved in mediating the regulation of hypoxically induced and Co^2+^-inducible genes (Figure 5B). These results suggest that the Co^2+^-inducible oxygen regulatory pathway plays only a minor role in mediating oxygen sensing and regulation.

MEDUSA identified the regulators that may mediate the regulation of oxygen-regulated genes that were affected at the early stage (1.5 hours) of anaerobic growth (Figure 5C and 5D) in cells bearing the Hap1 expression plasmid (*HAP1*), finding some regulators common to both time points and some specific to early or late stages. The results from analysis of both target genes (Figure S7) and regulators (Figure 5C and 5D and Figure S5) suggest that there was a significant switch in the regulatory and expression programs in the cells as anaerobic condition prolonged.

**Figure 6:**
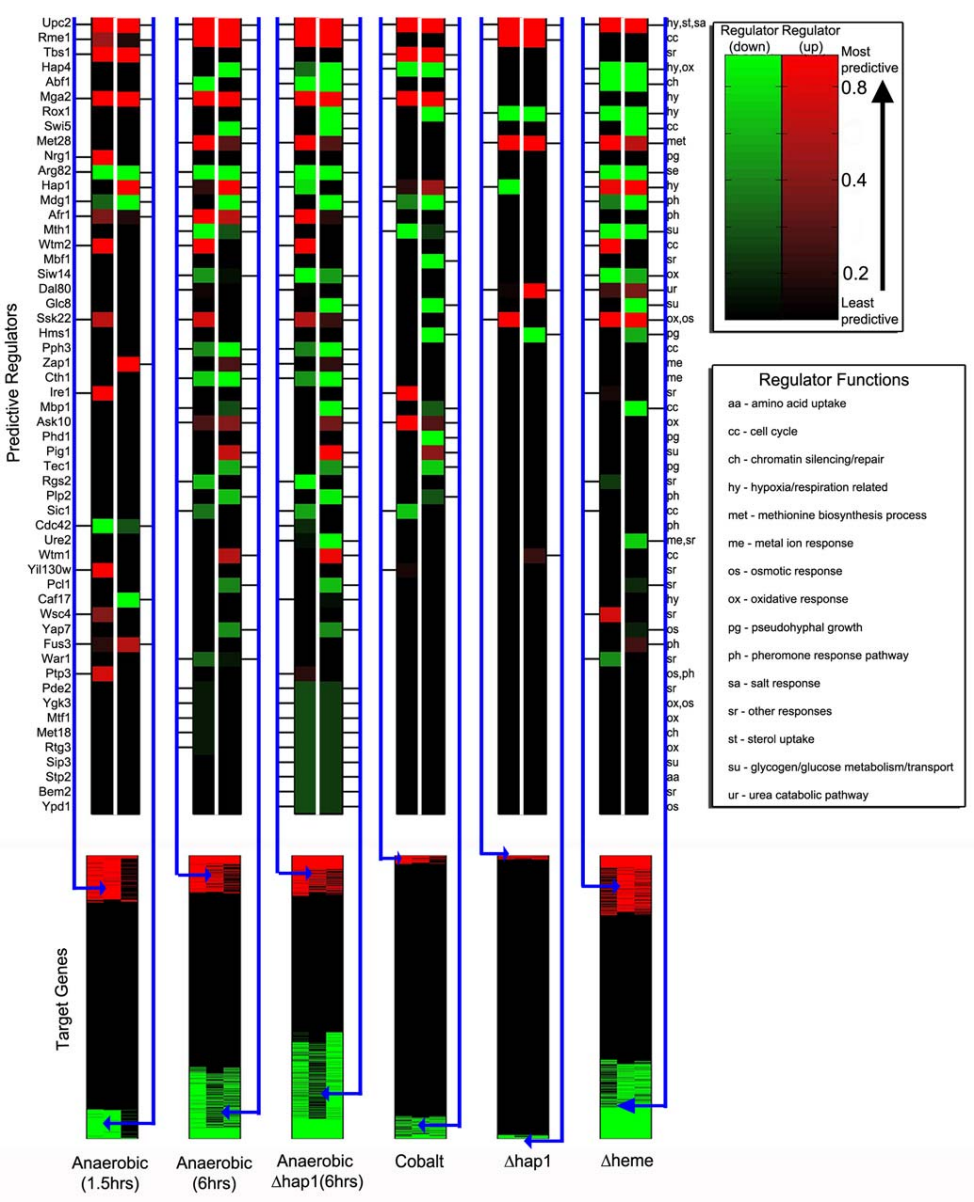
A global read-out of the oxygen regulatory network learned by MEDUSA. By applying margin-based scoring to the full list of potential regulators for the up- and downregulated target genes in each experimental condition, we identified 54 predictive regulators in the oxygen regulatory network. For each condition, we show the state of the regulator in red (upregulated) or green (downregulated), where the brightness of the color indicates the significance of its contribution to up or down predictions for the targets, based on normalized margin score. Significance of the regulators to the up-regulated targets is shown in the left half of the column, while contribution to the down-regulated targets is shown in the right half. Some regulators contribute significantly to the prediction of both up- and down-regulated targets within a condition due to indirect regulation (e.g., a transcriptional activator that controls a repressor), combinatorial effects, and promoter sequence information. Regulators are ranked from top to bottom in order of overall predictive significance across experiments, computed by taking the larger of the normalized margin scores for up and down targets in each experiment and then averaging across experiments. The functional category for each regulator is indicated by an annotation given at the right of the figure and explained in the legend.

To display the statistical importance of various regulators in the global oxygen and heme regulatory network, we summarized our results by using a global regulatory map (Figure 6). Figure 6 illustrates the significance of the regulators for predicting the up or down regulation of target genes under the tested six different experimental conditions, ranked by margin score. Several previously characterized regulators that are known to be important for oxygen and/or heme regulation, including Upc2, Rox1, Mga2, Hap4, and Hap1 [2], [27], [31]–[34], [46]–[51], ranked highly in this global regulatory map. Among the most significant regulators, six were previously known to be important for hypoxia response or oxygen regulation (Figure 6). Seven regulators known to be involved in cell cycle were identified by MEDUSA in this network. Intriguingly, six regulators known to be involved in pheromone response were identified (Figure 6). Likewise, several regulators known to regulate osmotic, salt and pseudohyphal growth were also identified. These results suggest that oxygen and heme regulation may share many regulators with pheromone and other signaling pathways. However, the regulators that are involved in general stress responses, such as Msn2, Msn4, and Hsf1 [52]–[55], were not identified by MEDUSA as significant regulators.

Figure 6 includes a total of 54 regulators identified by margin score for up/down regulated gene sets. We quantified the statistical significance of these regulators by computing an empirical null distribution for the margin score and then estimating *p*-values for each regulator/gene set (see Figure S10 and Figure S11 for details). We then used a Benjamini-Hochberg procedure [56] to estimate the false discovery rate (FDR) associated with various *p*-value thresholds (Figure S12). We find that if we set the *p*-value threshold so that all 54 regulators satisfy the significance threshold for at least one gene set, then our estimated FDR is less than .04.

### Experimental Data Measuring the OLE1 Promoter Activity Confirms Novel MEDUSA Regulators

To experimentally test specific regulators identified by using MEDUSA, we examined the *OLE1* promoter, because this promoter has been well characterized previously [31],[39],[57]. The full-length *OLE1* promoter-*lacZ* reporter activity is strongly induced by hypoxia [39]. By applying margin-based scoring to a set of previously identified *OLE1*-like genes [31],[39],[57], we extracted significant *OLE1*-specific motifs and regulators under hypoxia. Among the significant motifs, we found LORE (low oxygen response element), which has been experimentally determined [39] and is known to be the Mga2 binding site [31], as well as the binding sites for Hap1 and Aft1/2, which are also known to bind *OLE1* [58].

**Figure 7:**
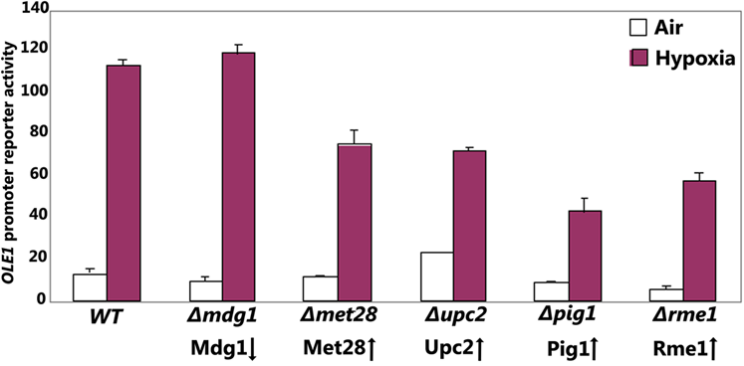
Experimental confirmation of the oxygen regulators identified by MEDUSA. MEDUSA identified Mdg1, Met28, Upc2, Pig1 and Rme1 as specific regulators of the hypoxia-inducible *OLE1* gene. To detect the effects of these regulators on the *OLE1* gene, the full-length *OLE1* promoter*-lacZ* reporter [39] was transformed into the wild type or mutant cells with one of the indicated genes deleted. β-galactosidase activities were measured in cells grown in air or in hypoxic chamber. Data plotted here are averages from at least three independent transformants. The arrows indicate the effects of hypoxia on the expression levels of Mdg1, Met28, Upc2, Pig1 and Rme1. That is, Mdg1 was downregulated whereas the rest were upregulated in hypoxic cells.

We then used margin-based scoring for regulators to identify Mdg1, Met28, Upc2, Pig1 and Rme1 as potential regulators for the *OLE1* promoter. Only Upc2 was previously known to be involved in oxygen regulation. The expression of all these regulators but Mdg1 was upregulated by hypoxia. Note that the MEDUSA model does not assert that these regulators directly bind the *OLE1* promoter but does predict that they regulate *OLE1* expression, perhaps through indirect interactions. Conceptually, the margin score for a regulator is similar to “knocking out” the regulator from the regulatory program and computing whether the effect is predicted to be significant for specific targets and conditions (see Methods). This connection suggests a direct approach for validation of these regulators using the corresponding deletion mutants. Namely, to determine the effects of these regulators on the *OLE1* promoter-*lacZ* reporter activity, we measured β-galactosidase activities in wild type and mutant cells with one of the regulator genes deleted (Figure 7). Except for *Δmdg1* cells, the reporter activity in the hypoxic mutant cells were all reduced, compared to that in wild type cells (Figure 7). Because hypoxia suppressed Mdg1 expression, indicating its negative role in *OLE1* induction, it is conceivable that its deletion would not affect the reporter activity in hypoxic cells. In contrast, because hypoxia induced the expression of other regulators, indicating their positive role in *OLE1* induction, their deletion would cause the reporter activity to decrease in hypoxic cells. Deletion of *MET28*, *UPC2*, and *PIG1* also significantly reduced the fold induction of the *OLE1* reporter activity by hypoxia (Figure 7). These experimental results strongly support the power of the MEDUSA analysis to predict regulators for specific targets.

### MEDUSA Reveals More Detailed Regulatory Information Than Standard Computational Analysis

We further analyzed whether MEDUSA could correctly extract regulatory information about a known functional regulon, and we compared the results of MEDUSA analysis with AlignACE [59],[60], a standard motif algorithm. We then did a global comparison of transcription factor binding motifs attributed by MEDUSA and AlignACE to the 16 expression signatures in our dataset.

The functional regulon we examined was signature 16, one of the smaller expression patterns consisting of a set of 34 *HAP1*-dependent genes that are strongly suppressed in all conditions including the *Δhap1* experiment. These genes (such as the *COX* and *QCR* genes) are involved in aerobic respiratory processes (1e-14), electron transport (4.37e-14) and heme-dependent oxidoreducatase activity (5.77e-14). Starting with the global MEDUSA regulatory program, we extracted all sequence motifs and regulators with positive margin score for this set of genes and further ranked them based on the number of target genes they were predicted to regulate.

We first considered transcription factors that were high ranking by both regulator and motif criteria. Namely, they were high scoring regulators based on their mRNA expression states and their binding site motifs were discovered by MEDUSA and ranked as significant and frequent for the 34 genes in the signature. This set of transcriptional regulators consisted of Hap1, Mot3, Ace2, Mac1, Msn2, Ste12, Gcn4, Pho4 and Hap2/3/4. MEDUSA also ranks the Hap1 and Hap4 ChIP-chip occupancy profiles as highly significant features for these genes. By examining the literature, we found support for many of these transcription factors regulating at least several of the target genes in this set. For example, *CYC1*, *CYC7* and *CYT1* are known to be directly regulated by Hap1 [61]. Hap2/3/4 is known to directly affect expression of *COX4*, *COX5*, *COX6*, *CYC1* and *CYT1* [61]. Mot3 is known to directly regulate *CYC1* expression [62]. *FRE1* and *CTR3* are known to be regulated by Mac1 [61],[63]. Upc2 and Mga2 are also important hypoxia regulators, and MEDUSA identified their binding sites as high scoring motifs for smaller subsets of genes within the signature. MEDUSA also identifies Abf1 as a significant regulator through its mRNA expression, but the sequence motif corresponding to the Abf1 binding site and the Abf1 ChIP-chip data have low margin scores. Some of the *COX* genes are known to be regulated by Abf1 in other conditions [61], and the Abf1 binding site is present in several of the genes. In this case, our MEDUSA analysis suggests mixed evidence for Abf1 as a transcriptional regulator of the regulon, and it is possible that under hypoxia other regulators dominate.

As a comparison, we also used AlignACE to find overrepresented sequence motifs in the promoter regions of this gene set. Since the signature is small and represents a true functional regulon, it provides an ideal case for traditional motif discovery algorithms. Using the motif discovery program in the most permissive way (that is, without enforcing any significance threshold on the motifs), AlignACE was only able to find significant hits for the binding sites of Cha4, Hap1, Ace2 and Gcn4. For Hap2/3/4, the MAP score (3.4) had very low statistical significance, even though the motif is very abundant in this gene set (46.1%) and is known to regulate most of these genes [61]. Also, AlignACE was not able to identify more subtle context-specific regulators such as Mot3, Mac1, and Mga2, which are known to regulate these genes.

**Figure 8:**
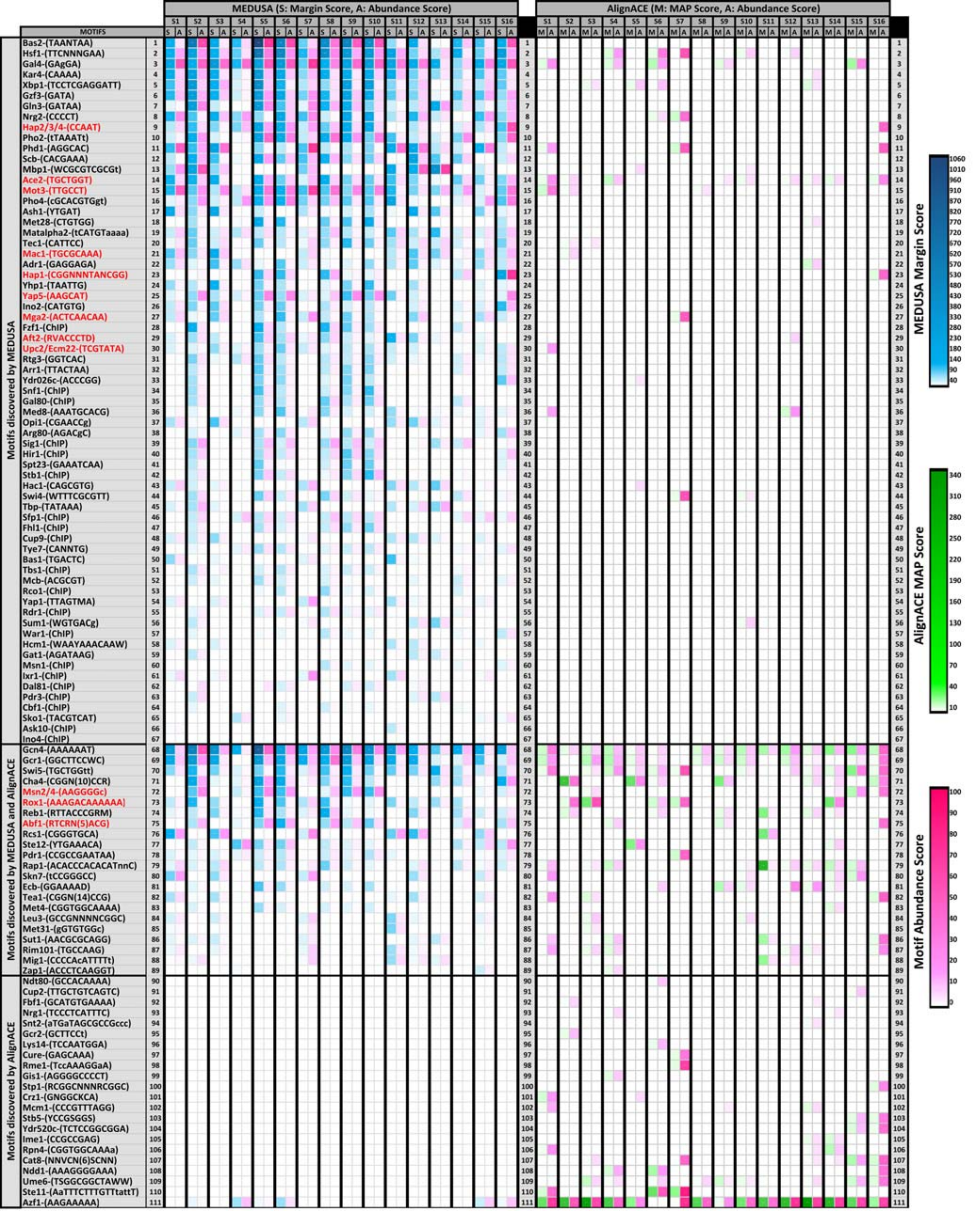
Comparison of significance and abundance of motifs learned by MEDUSA and AlignACE for the 16 expression signatures identified in the dataset. Each row in the table represents a motif found by MEDUSA only (top section), by both MEDUSA and AlignACE (middle section), or by AlignACE only (bottom section). The first column describes the motif by the name of the corresponding transcription factor followed by the consensus motif sequence. Some transcription factor names are followed by ‘ChIP’, indicating that these are significant ChIP-chip occupancy features identified by MEDUSA. Motif descriptions highlighted in red indicate transcription factors that are specifically known to have an important function in hypoxia. The remainder of the table shows MEDUSA (left section) and AlignACE (right section) results for each signature (S1 to S16), represented by a pair of columns scoring motifs by statistical significance (left column in each pair) and abundance within the set of genes making up the signature (right column in each pair). For statistical significance scores, columns labeled ‘S’ represent the margin scores (in shades of blue) assigned by MEDUSA, and columns labeled ‘M’ represent the maximum a posteriori (MAP) scores (in shades of green) assigned by AlignACE. In both cases, dark shades indicate higher statistical significance. The columns labeled ‘A’ show the percentage abundance scores of the motifs in each of the signatures. For AlignACE, the abundance score of a motif simply reflects the ratio of the number of genes in each cluster whose promoters contain the motif, to the cluster size. For MEDUSA, it refers to the ratio of the number of genes in each cluster for which the motif contributes positively to the margin score, to the size of the cluster. A motif could be present in the promoter of a gene but not identified as significant by MEDUSA. In such cases, the motif does not contribute to the MEDUSA abundance score. Dark shades of pink indicate strong abundance scores.

Figure 8 shows a comprehensive comparison of MEDUSA to AlignACE motif discovery results across all 16 signatures. We used AlignACE with default settings on 1000 base pair promoter sequences of genes belonging to each signature and used AlignACE's maximum a posteriori (MAP) scores to rank their statistical significance. We also defined the abundance score for each motif as the fraction of promoters that were found to have the motif. Similarly, we used margin scoring to identify significant MEDUSA motifs for each of the signatures, reporting only those motifs with a positive margin score. For MEDUSA, we defined the abundance score for a motif as the fraction of promoters in the signature set that were found to have the motif based on the tree structure of the learned model. In order to compare the two methods, we report in Figure 8 only those motifs that matched known transcription factor binding sites PSSMs in TRANSFAC, SCPD or YPD (using Kullback-Leibler divergence to compare motifs [8]) or matched consensus sequences found by MacIsaac et al. [64]. (A separate comparison of MEDUSA motifs to MacIsaac et al. motifs alone appears in Figure S9.) If multiple motifs were found to be strong matches to the same known binding site, we reported the one with the highest statistical score. In total, we matched 111 motifs found by either or both methods to known binding sites, and we sorted these motifs into 3 categories based on the difference between the cumulative margin score and cumulative MAP score across all the signatures. The first set consists of 67 motifs identified by MEDUSA but not by AlignACE; the second set consists of 22 motifs that are identified by both MEDUSA and AlignACE; and the third set consists of 22 motifs that are identified by AlignACE but not MEDUSA. In Figure 8, the motifs highlighted in red are binding sites of transcription factors known to play a key role in hypoxia-related conditions. MEDUSA is able to identify a number important hypoxia-related transcription factor binding sites, including Hap1 (CGGnnTAnCGG), Hap2/3/4 (CCAAT), Mga2 (ACTCAACAA), Upc2/Ecm22 (TCGTATA), Ace2 (TGCTGGT), Mot3 (TTGCCT), Mac1 (TGCGCAAA), Aft2 (RVACCCTD), Msn2/4 (AAGGGGc), Rox1 (AAAGACAAAAAA) and Abf1 (RTCRnnnnnACG). Among these, AlignACE is able to identify Rox1, Msn4 and Abf1, and it finds the Hap1 and Hap2/3/4 binding sites only for a single signature (signature 16). Moreover, none of the motifs exclusively identified by AlignACE are known to have any role in the hypoxia-related conditions. In particular, the top scoring AlignACE motif is a low complexity motif (AAAAAAAA) that matches the Azf1 binding site. These results show that MEDUSA outperforms AlignACE in finding relevant sequence motifs for our dataset.

## Discussion

In this study, we applied a novel machine learning algorithm to learn regulatory programs underlying oxygen regulation and heme regulation. This algorithm uses experimental data, including microarray gene expression data, promoter sequence, and ChIP-chip data, without introducing prior assumptions such as presuming a cluster structure in gene expression data. The results from our analysis show that the MEDUSA algorithm can provide important, unbiased information about global regulatory programs. MEDUSA identifies many DNA sequence motifs important for oxygen and heme regulation (Figure 8). Further, experimental data from measuring *OLE1* promoter activity confirms the specific predictions made by MEDUSA (Figure 7).

Finally, a comprehensive comparison with a traditional “cluster-first” motif discovery approach demonstrated that MEDUSA is more successful at identifying binding site motifs relevant to oxygen regulation (Figure 8).

MEDUSA identifies many regulators previously known to be involved in this system. For example, MEDUSA identifies Upc2, Mga2, and Hap1 [2],[32],[33],[37],[46],[48],[65] as important regulators in oxygen regulation (Figure 5). Further, MEDUSA predicts many new regulators of oxygen and heme regulation, such as Pph3, Bem2, and Pcl1. In support of the regulatory network identified by MEDUSA, several identified regulators are known to interact with each other. For example, Mbp1 and Ure2 are known to coexist in one complex, and the MAP kinase kinase kinase Ssk22 acts upstream of Mbp1. Pph3 and Bem2 are known to coexist in one complex, and both likely mediate the regulation of both hypoxically induced genes and oxygen-induced genes in *Δhap1* cells (Figures 4A, 5A, and 6). Wtm1 and Afr1, which are known to coexist in one complex, act in concert to promote oxygen regulation in wild type *HAP1* cells (Figures 4A and 6). Likewise, Ire1, which acts upstream of Rgs2, may act with Rgs2 to mediate the regulation of heme-suppressed genes (Figures 5A and 6).

The purpose of achieving high prediction accuracy on the test data is to confirm that the identified regulators are statistically important predictors for the regulation of target genes. The number of significant regulators identified by MEDUSA is much smaller than the number of regulators whose expression is changed in a specific experiment. For example, in cells bearing the Hap1 expression plasmid (*HAP1*), we identified 18 significant regulators that may mediate the regulation of the oxygen-induced genes (Figure 4A), out of 98 regulators whose mRNA levels were significantly altered in the experiment. This dramatic filtering is achieved by three aspects of our computational approach. First, we require that regulators control their putative targets through shared motifs in the promoter sequences. Second, we train on examples from multiple experimental conditions. If a regulator cannot be associated with a binding site motif through which it contributes to target gene regulation in a consistent way across multiple conditions, it will not be selected by the algorithm. Finally, we use a novel margin-based score to identify the most significant regulators for specific conditional and gene sets. This filtering represents an important improvement over simply correlating expression levels of regulators with those of target genes.

It is important to note that the MEDUSA analysis did not identify the regulators that mediate stress responses, such as Msn2, Msn4, Tpk1, Usv1, Yap1, and Hsf1 [22], [52]–[55],[66],[67], although motifs for some of these regulators are identified in the promoters of target genes. In some aspects, anaerobic and heme-deficient conditions exhibit certain characteristics of stress responses. As such, certain genes induced by stress, such as genes involved in ribosome synthesis, were induced by anaerobic and heme-deficient conditions (see Figure S8). However, the regulatory network mediating oxygen and heme regulation is clearly different from the general stress response network. The most significant regulators in the oxygen and heme regulatory network are not those involved in general stress responses. Interestingly, however, this oxygen and heme regulatory network shares many regulators with other signaling pathways, such as pheromone signaling and osmotic responses.

Our analyses suggest a remarkable flexibility of the oxygen and heme regulatory network. For example, in the absence of Hap1, certain new regulators, such as Glc8 and Mbp1 and Ure2, along with some of the regulators acting in wild type *HAP1* cells, appear to be recruited to mediate oxygen regulation to substitute Hap1 (Figure 6). Another feature of the oxygen and heme regulation network is its complexity. Although several previously known oxygen and heme regulators, including Hap1 and Mga2 [32],[46], are confirmed to be important in oxygen and heme regulation by our analysis, many other regulators appear to play important roles in global oxygen and heme regulation. Through biochemical validation of the predicted regulators for the *OLE1* promoter, we have taken the first step in confirming the novel components of the oxygen regulatory network as predicted by MEDUSA. While much experimental work remains to be done, we are encouraged by MEDUSA's success in generating condition- and target-specific hypotheses that we were able to validate experimentally.

MEDUSA's “cluster-free” approach has the advantage that it can still be effective for small expression datasets, where clustering may only generate large and functionally heterogeneous gene sets. Moreover, clustering and most “module” learning approaches rely on the static assignment of genes to clusters across all experiments in a dataset, which may oversimplify coregulation relationships between genes. MEDUSA models condition-specific regulation in a more flexible way that avoids the cluster assumption. However, methods that produce sets of clusters or modules are more familiar and easier to visualize than MEDUSA's gene regulatory programs, and MEDUSA analysis requires an interpretation step to reveal detailed information for specific conditions or sets of genes. In the present work, we used margin scoring to extract significant regulators for the set of induced/suppressed target genes in each condition and significant motifs associated with genes belonging to expression signatures. This analysis gave a convenient summarization of our results, but more general kinds of statistical post-processing are possible and could be more informative. MEDUSA is well suited to a perturbation dataset, where the set of regulators exhibit diverse expression signatures across conditions. In a dataset where many of the regulators are highly correlated, such as in a short time series, there may not be enough information in the discretized regulator expression profiles for MEDUSA to resolve condition-specific regulators. More generally, MEDUSA incurs some loss of information by discretizing gene expression data prior to training. Extending MEDUSA to handle real-valued regulator and target gene expression levels, for example through a regression formulation, might address this limitation, but it could also introduce too much noise and lead to over-fitting. Further investigation is needed to determine whether such extensions to the MEDUSA algorithm will lead to greater biological insight.

## Methods

### Yeast Cell Growth and Treatment

Yeast strains used were L51 (MATa, *ura3-52*, *leu2-3*, *112*, *his4-519*, *ade1-100*, *trp1::HisG*, *hap1::LEU2*) and MHY100 (MATa, *ura3-52*, *leu2-3*, *112*, *his4-519*, *ade1-100*, *hem1-Δ100*) [68]. L51 was used for studies of oxygen regulation, and MHY100 was used for studies of heme regulation. To avoid variations from the differences accumulated after many generations of growth of strains, we transformed the L51 strain with the *HAP1* gene deleted for studies of Hap1 function. Hap1 protein was expressed in L51 cells by transforming an ARS-CEN plasmid bearing the complete *HAP1* genomic sequence [69]. For comparison with cells without Hap1 expressed, an empty vector was transformed into L51 cells. The use of Hap1 expression plasmid generated much more reproducible results than the use of different strains. Yeast cells with or without Hap1 expressed grew at similar rates under both anaerobic and aerobic conditions.

We chose to use a low oxygen level (∼10 ppb) in this study, in order to identify oxygen-regulated genes. Previous studies have shown that most, if not all, oxygen-regulated genes are affected at low concentrations, but some genes are not affected at higher oxygen levels (for example, >1 ppm) [26],[45]. Anaerobic (∼10 ppb O2, measured by using an oxygen monitor and confirmed by CHEMetrics oxygen kits) growth condition was created by using an anaerobic chamber (Coy Laboratory, Inc.) and by filling the chamber with a mixture of 5% H_2_ and 95% N_2_ in the presence of palladium catalyst [45]. The oxygen level in the chamber was monitored by using the Model 10 gas analyzer (COY laboratory Inc). H_2_ was filled to keep the measured oxygen level at zero. The precise level of oxygen was further measured by using rhodazine kit (K-7511) with MDL at 1 ppb, and a range of 0–20 ppb (http://www.envco.info/prod.php?product_id=469). L51 cells bearing the Hap1 expression or empty vector were grown under normoxic or anaerobic conditions for 1.5 or 6 hours. The UAS1/*CYC1-lacZ* reporter plasmid [70] was transformed into yeast cells to confirm the expression of Hap1 and the oxygen levels. Cells were grown in yeast synthetic complete media. Co^2+^-induced cells were grown in the presence of 400 µM cobalt chloride for 6 hours, as described previously [31],[32]. MHY100 cells were grown in medium containing 2.5 µg/ml (heme-deficient) or 250 µg/ml (heme-sufficient) 5-aminolevulinic acid [68]. For RNA preparations, yeast cells were inoculated so that the optical density of yeast cells was in the range of 0.8–1.0 immediately before the collection of cells.

### RNA Preparation and Microarray Gene Expression Profiling

RNA was extracted from yeast cells exactly as previously described [71]. RNA samples were prepared from 8 different experimental conditions: (1) L51 yeast cells bearing the Hap1 expression plasmid maintained under aerobic conditions, (2) L51 yeast cells bearing the empty expression plasmid maintained under aerobic conditions, (3) L51 yeast cells bearing the Hap1 expression plasmid maintained under anaerobic conditions for 1.5 hours, (4) L51 yeast cells bearing the Hap1 expression plasmid maintained under anaerobic conditions for 6 hours, (5) L51 yeast cells bearing the empty expression plasmid maintained under anaerobic conditions for 6 hours, (6) L51 yeast cells bearing the Hap1 expression plasmid in the presence of 400 µM cobalt chloride for 6 hours, (7) MHY100 cells grown in medium containing 250 µg/ml (heme-sufficient) 5-aminolevulinic acid, and (8) MHY100 cells grown in medium containing 2.5 µg/ml (heme-deficient) 5-aminolevulinic acid. For each condition, three replicates were generated by preparing RNA samples from three batches of independently grown cells. Microarray expression analyses were performed by using three batches of replicate RNA samples. The quality of RNA was high as assessed by measuring absorbance at 260 and 280 nm, by gel electrophoresis, and by the quality of microarray data (see below).

The synthesis of cDNA and biotin-labeled cRNA were carried out exactly as described in the Affymetrix GeneChip Expression Analysis Technical Manual (2000). The yeast Saccharomyces cerevisiae genome 2.0 arrays were purchased from Affymetrix, Inc. Probe hybridization and data collection were carried out by the Columbia University Affymetrix GeneChip processing center. Specifically, the Affymetrix GeneChip Hybridization Oven 640 and the next generation GeneChip Fluidics Station 450 were used for hybridization and chip processing. Chip scanning was performed by using the GeneChip scanner 3000. Initial data acquisition analysis was performed by using the Affymetrix Microarray suite. By using GCOS1.2 with the advanced PLIER (probe logarithmic intensity error) algorithm, we calculated and examined the parameters reflecting the image quality of the arrays. Arrays with a high background level in any region were discarded and replaced. The average noise or background level was limited to less than 5%. The average intensity for those genes judged to be present was at least 10-fold higher than those judged to be absent. Also, arrays that deviated considerably in the percentage of present and absent genes from the majority of the arrays were replaced. Arrays with a β-actin 3′/5′ ratio greater than 2 were replaced.

### Normalization of Microarray Data

For each microarray, we converted the .DAT image files into .CEL files using the Affymetrix GCOS software. These raw .CEL files were further processed into expression values using the RMA express software by Bolstad [72] (Dataset S1). This software uses the robust multiarray average method by Irrizary et al. [73], which involves a background correction and a quantile-based normalization scheme.

### Discretization of Microarray Data

Each of the knockout, stress, or perturbation microarray experiments was compared to a corresponding reference microarray as shown in Figure 2B. The expression fold-changes (Dataset S3) were converted to *p*-values using an intensity-specific noise model obtained from replicate data. We used the methodology outlined in Stolovitzky et al. [74] to generate separate empirical noise models for each of the reference conditions. The fold-changes were then discretized into +1, 0 or −1 labels using a *p*-value threshold of 0.05. A label of +1 (−1) indicates up-regulation (down-regulation) beyond the threshold level of noise.

Several genes have multiple probes on the microarrays. In such cases, we discretized each probe reading independently and used a majority vote over the +1 and −1 discretized values to obtain a final label for the gene. In cases where a majority vote was not possible (due to equal number of probes with +1 and −1 values), we used the label corresponding to the reading with the lowest *p*-value. All the replicate experiments were used as input to MEDUSA. However, in order to remove inconsistency, for each gene, we further filtered out expression values that did not agree with the consensus label (+1 or −1) across replicates of a particular experimental condition. The discretized gene expression data is presented in Dataset S2. Dataset S4 lists the sets of differentially expressed genes in each of the experimental conditions.

### Database Annotations

We obtained gene annotations and functional associations from Saccharomyces Genome Database (SGD, ftp://ftp.yeastgenome.org/yeast/, Jan 2006). The gene ontology tree structure was downloaded from the Gene Ontology Consortium [75]. To identify statistically enriched terms associated with sets of genes, we calculated *p*-values using the cumulative hypergeometric null distribution on the basis of the number of genes in the set, the number of genes in that set that are annotated with each GO term, and the number of genes in the genome that are annotated with that GO term. We then filtered terms using 1e-5 as a threshold.

### Candidate Regulators

We used a set of 507 regulators consisting of 240 known and putative transcription factors and 267 known and putative signaling molecules such as kinases, phosphatases and receptors. This set includes 466 regulators from Segal et al. [22] and 9 generic (global) regulators from Lee et al. [76].

### Promoter Sequences

For the motif discovery phase in MEDUSA, we used 1000 bp nucleotide sequences upstream of the TSS of all *S. cerevisiae* genes that we obtained from the Saccharomyces genome database (SGD, ftp://ftp.yeastgenome.org/yeast/, Jan 2006). We scanned these sequences for all occurring *k*-mer motifs (*k* = 2, 3, … , 7) as well as 3-3 and 4-4 dimer motifs allowing a middle gap of up to 8 bp. We restricted the set of all dimers to those whose two components have specific relationships, consistent with most known dimer motifs: equal, reversed, complements, or reverse complements. The MEDUSA algorithm uses an information-theoretic, hierarchical agglomeration scheme to learn position-specific scoring matrices (PSSMs) from *k*-mers and dimers occurring in the promoter sequences. We generated a set of 450 PSSMs in the first pass of MEDUSA on our dataset.

### Chromatin Immunoprecipitation Microarray Data

Harbison et al. [77] use genome-wide location analysis, based on modified chromatin immunoprecipitation (ChIP), to identify genomic binding sites for 203 transcription factors in living yeast cells under 13 diverse environmental conditions, using upstream regions of approximately 6000 genes. For each genomic region, the transcription factor occupancy is reported as the log intensity ratio of the IP-enriched channel versus the genomic DNA channel, and a single array error model [76] is used to assign *p*-values to these measurements. In the second pass of the MEDUSA algorithm, we used the ChIP data to obtain additional binary features by thresholding the *p*-values, augmenting each target gene's motif vector by ChIP occupancy features corresponding to set of transcription factors. We tried different thresholds of 0.001, 0.05 and 0.1 (results not shown) and found the best prediction accuracy with a *p*-value threshold of 0.1. Although Harbison et al. [77] suggest a stricter *p*-value threshold, their suggestion is based on minimizing false positives, while in MEDUSA, we use these binary features to define abstaining weak rules for boosting (see below). Theoretically, weak rules need only give a small advantage over random guessing to satisfy the assumptions of boosting. In this context, false negatives are more of a concern, since rules with very sparse hits lead to slow and modest improvement in prediction accuracy. Moreover, other studies suggest that low affinity binding in ChIP experiments may indeed be functional, based on sequence conservation evidence [78].

### Expression Signatures

We used a two-phase procedure to partition the genes into expression signatures for an initial qualitative view of the dataset.

The first phase was used to identify the number of unique signatures. We first averaged the expression data for each gene in each experimental condition over all replicates. We then discretized this expression data into 2 levels (+1/−1) based on the sign of the foldchange in expression. We grouped genes into sets of unique patterns across all experiments. We then averaged the real-valued expression data for all genes belonging to each pattern to obtain a “mean expression signature”. Patterns with small support (<10 genes) were hierarchically merged with their nearest pattern until there were no patterns with <10 genes. The nearest neighbor was determined using a square-euclidean distance metric over the mean expression signatures for each pattern. This procedure grouped the 3358 significantly expressed genes to be grouped into 16 sets.

The second phase was used to refine the clustering. We reclustered the genes into 16 expression signatures using the *k*-means clustering algorithm. We used the square-euclidean distance metric over the real-valued expression data. In an attempt to avoid local minima, we repeated the clustering procedure 10 times with different starting points. The initial candidate genes for cluster centroids were obtained by randomly sampling the 16 gene sets identified in the first phase. The final expression signatures were calculated by taking a majority vote over the 3-level (+1/0/−1) discretized expression for all genes in each gene set.

### Nearest Neighbor Methods

We compared MEDUSA to a simple correlation-based method, where we predict a gene's held-out expression levels based on the “nearest regulator” to its training set expression levels. We calculated prediction accuracies for 10-fold cross validation using held-out examples and by grouping replicates in the same fold. To avoid any bias, we reported accuracies on target genes excluding the 503 regulators. As in our main experiments, we performed 10-fold cross-validation, and for each gene represented in the test set, we considered its expression profile when restricted to examples (i.e., experiments) in the training set and found the best-correlated regulator across these experiments. The discrete expression level of this regulator was then used to predict up/down expression in experiments held out for this gene. As a similarity metric, we used the normalized Hamming distance (excluding baseline examples) for discretized expression data, where the inclusion/exclusion of baseline examples was chosen in order to report the better results. In cases where multiple regulators were equally distant from a target gene, we randomly selected one amongst them as the “nearest regulator”.

### MEDUSA Software

An open source matlab implementation of the MEDUSA algorithm is freely available for academic users and downloadable from http://cbio.mskcc.org/leslielab/software/medusa.

### The MEDUSA Regulation Model

The MEDUSA algorithm models the control logic of transcriptional regulation in the form of an alternating decision tree (ADT). An ADT is a generalization of a decision tree that consists of alternating layers of decision nodes and prediction nodes. Decision nodes ask yes/no questions based on particular features. Prediction nodes contain a real-valued score contribution associated with the yes or no answer. Unlike regular decision trees, which make yes/no predictions, ADTs generate real-valued prediction scores whose sign gives the up/down prediction and whose size gives a measure of confidence in that prediction. ADTs do not necessarily correspond to a nested set of binary partitions of the example space like regulator decision trees; that is, there can be more than one decision node beneath a prediction node, and more than one of these decision node conditions can be true for a given example.

Given the promoter sequence of a gene and the expression level of the regulators in an experiment, the MEDUSA ADT predicts up/down regulation of the gene in this experiment using yes/no questions of the form, “Is motif *μ* present in the upstream region of the gene and is the state of regulator *ρ* up (or down) in that experiment?”, in the decision nodes. If the answer is “yes”, we add the associated real value score contribution to the overall prediction score for the example, and we continue down to the next decision node; for greater interpretability, we abstain if the answer to the decision node condition is “no”; that is, we contribute zero to the prediction score. To compute the prediction score for a gene-experiment example, we start at the root node and recursively check which decision nodes we can pass through by answering “yes” to the condition, working from the top to the bottom of the ADT; the final prediction score is the sum of all the score contributions in all paths in the ADT that we visit in this process.

More formally, the ADT takes the feature representation *x_ge_* of the gene-experiment pair (*g*,*e*)—namely the promoter sequence for gene *g* and the vector of expression states of regulators in experiment *e*—and computes a prediction score 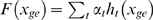, a weighted sum of *weak rules h_t_*, where *t* is an index over *paths* in the tree. If *c* is a boolean condition, then define *h*[*c*] to be the weak rule that evaluates to 1 if the *c* is true and to 0 if *c* is false, i.e., *h*[*c*](*x_ge_*) = 1(0) exactly when *c* = **T(F)** for example *x_ge_*. If the *t*-th path has length *l* and corresponds to a sequence of decision nodes with conditions *c*_1_,*c*_2_,…,*c_l_*, then the weak rule *h_t_* = *h*[*c*_1_ ∧ *c*_2_ ∧ … ∧ *c_l_*] corresponds to the *conjunction* of these conditions. The weight *α_t_* is the score contribution in the prediction node for the lowest (depth l) decision node. The sign of the prediction score *F* (*x_ge_*) yields the up/down prediction for the example.

### Learning Predictive Models

We learn the gene regulation program *F* (*x_ge_*) in two phases. In the first phase, we input discretized regulator and target gene expression data along with target gene promoter sequences to the core MEDUSA algorithm to discover DNA motifs and assemble an initial ADT model. The core algorithm is described below. In the second phase, we take the motif hits learned in the first ADT, together with discretized target and regulator expression data and ChIP chip occupancy data, to learn a stabilized ADT, as described in the next section.

To learn the ADT in the first training phase, we use a training set {*x_ge_*, *y_ge_*} of labeled examples, where *x_ge_* is the feature representation of the target gene-experiment pair (*g*,*e*) (promoter sequence of *g* and vector of expression states of regulators in experiment *e*), labeled as *y_ge_* = +1 if the gene is up-regulated in the experiment and *y_ge_* = −1 if it is down-regulated. Examples that are not significantly differentially expressed (“baseline”) are excluded from the training set. MEDUSA uses a supervised learning algorithm based on boosting to iteratively build an alternating decision tree (ADT) that represents a gene regulation model for all target genes and experimental conditions. MEDUSA learns binding site motifs from the target gene promoter sequences while building the ADT, but the same ADT learning algorithm can be run using a fixed set of motifs as an input [40],[41]. In our hypoxia experiments, the training set consisted of 13,093 differentially expressed gene-experiment instances.

Boosting is a general machine learning algorithm for binary prediction problems. The basic idea is to iteratively apply a simple discriminative learning algorithm, called the weak learner, to different weightings of the same training set. At each iteration *t* = 1…*T*, boosting selects a weak rule *h_t_* that optimizes an exponential loss function for the current weights; then weights *w_ge_* are recalculated so that examples *x_ge_* that were misclassified by *h_t_* are more highly weighted. Finally, all of the weak rules are combined into a single strong rule using a weighted majority vote. Empirically, boosting often learns to make large-margin (confident) predictions on the training set, which is theoretically linked to its ability to obtain good generalization on test data even when the feature space is very high dimensional (that is, it avoids over-fitting the training data).

**Figure 9:**
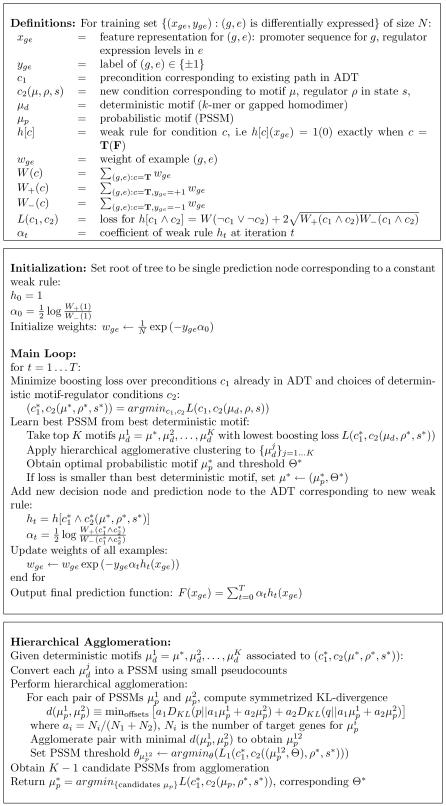
Pseudocode for the MEDUSA learning algorithm. The figure gives detailed pseudocode for the core MEDUSA algorithm which learns DNA motifs de novo from promoter sequences and assembles motifs and regulators into an alternating decision tree (ADT) for predicting up/down regulation of target genes.

Detailed pseudocode for the core MEDUSA algorithm is given in Figure 9. Briefly, each iteration *t* of boosting adds a new decision node—corresponding to a binding site motif *μ*, coupled with a regulator *ρ* whose state *s* (either up or down) helps predict up/down regulation of target genes—and a prediction node to a gene regulation model described by an ADT. Each motif is either a *k*-length sequence (“*k*-mer”), a dimer, or a PSSM. The weak rule *h_t_* defined by the decision node depends both on the motif-regulator condition that it tests and on the position at which it is placed in the ADT. We define the *precondition c*_1_ to be the conjunction of conditions in decision nodes along the path to the existing prediction node under which the new decision node is placed, and we write *c*_2_ = *c*_2_ (*μ*, *ρ*, *s*) as the condition tested in the new decision node. Then the corresponding weak rule is *h_t_* = [*c*_1_ ∧ *c*_2_], and the prediction node value *α_t_* can be computed from the weight of the correct and incorrect training predictions made by *h_t_* (see Figure 9). The motif *μ* added at iteration *t* is learned in two stages. First, the algorithm considers all deterministic motifs (*k*-mers or dimers) *μ_d_* and optimizes boosting loss over choices of preconditions *c*_1_ and new conditions *c*_2_ = *c*_2_ (*μ*, *ρ*, *s*), yielding optimal precondition 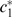, regulator 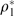 and state *s* ^*^. Second, candidate PSSMs are generated by considering the top-ranked deterministic motifs 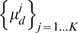 generated in the first stage and performing hierarchical agglomeration (see Figure 9). Optimizing boosting loss over candidate PSSMs and choices of thresholds for the log-odds score for each of these PSSMs yields an optimal probabilistic motif 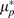 and threshold *θ* ^*^. This motif is used for the new decision and prediction nodes if its loss is better than the best deterministic motif 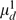.

We ran the core MEDUSA algorithm for 450 iterations to obtain a set of 450 PSSMs, their targets and thresholds along with an initial ADT. We decided on the stopping criterion based on the number of iterations required for the mean test-loss on cross-validated folds to plateau.

### Stabilized Predictive Models

In standard boosting, features that are correlated with the single best feature at a particular round of the algorithm are decorrelated in the next round of boosting and may fail to be captured by the model. While this possible consequence does not affect prediction accuracy, we may lose biologically important features that are correlated to the ones selected by the algorithm. To address this issue, we previously introduced a stabilized variant of boosting that can be used with ADT learning for modeling gene regulation [41]. The main idea of stabilized boosting is to allow a set of correlated features, rather than single features, to be included at nodes of the tree. We average the prediction of several weak rules in the case where the rules with smallest boosting loss are highly correlated with each other.

More precisely, at each iteration of ADT learning, we find the optimal precondition 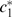 and new decision node condition 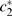, and we first decide whether to stabilize the node by looking for conditions correlated to 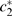. We stabilize only if the weak rule 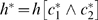 itself provides a significant advantage over random guessing, namely if
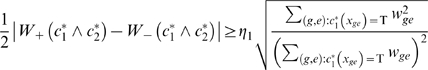
where *η*_1_ is a parameter and other notation is as defined in Figure 9. If this condition is satisfied, we look for additional conditions *c*_2_ such that the weight of the symmetric difference of the examples where conditions 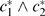 and 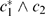 hold is sufficiently small, i.e.,
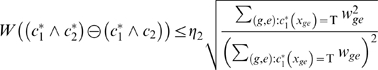
where *η*_2_ is a second parameter. We then average over the weak rules corresponding to 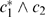 as follows. For a condition *c*, write the coefficient of the corresponding weak rule *h* as 
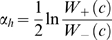
. For the set of weak rules 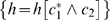, we define a new stabilized rule *h_avg_* that takes a robust majority vote over the set of weak rules as follows:
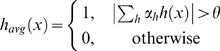
where we set 
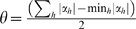
. In our experiments, we use *η*_1_ = *η*_2_ = 0.1. Theoretical motivation for the stabilized boosting equations can be found in our earlier work [41].

Stabilized boosting retains correlated features, so that in post-processing we obtain more stable ranked lists of regulator and motif features. The improved stability of the prediction tree allows us to perform detailed condition-specific post-processing of target gene sets.

For the second phase of training on the hypoxia data, we used the stabilized boosting algorithm again for 450 iterations to relearn the prediction tree using the motif hits learned in the initial MEDUSA run together with promoter occupancy data for transcription factors from ChIP-chip experiments. Here, we view a promoter sequence as either as bound by a transcription factor (TF) in the ChIP assay by thresholding on the *p*-value for the ChIP chip occupancy ratio. We note that the stabilized decision nodes in the ADT often contained a set of conditions involving a single motif paired with different regulators. This final model was used for all post-processing analysis (see Dataset S5).

### Feature Extraction and Significance

The *margin* of a gene-experiment example is given by *y_ge_F* (*x_ge_*), where *F* (*x_ge_*) is the real-valued score assigned to the example by the ADT and *y_ge_* is its binary label (+1 for up or −1 for down). The margin is positive if the ADT correctly predicts the example, and the size of the margin can be interpreted as the confidence of the prediction [79]. Motivated by theoretical work that links large margins on the training set with good generalization to test data, we developed a margin-based score to evaluate the importance of a given regulator to the overall predictive model. For a regulator *ρ*, we write *F_ρ_*_→0_ (*x_ge_*) to be the modified score that the ADT would assign a given gene-experiment example if we artificially set the regulator's expression level to baseline in the experiment. Equivalently, we could obtain the modified score for the example by eliminating all nodes containing *ρ*, and all subtrees rooted at these nodes, from the ADT, and then computing the prediction score. To assess the significance of *ρ* for a particular set of genes *G* in a particular set of experiments *E* in the training set, we computed the following margin-based score:
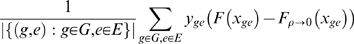



This score will again be positive if regulator *ρ* contributes on average to correct predictions, and the size of the score can be used to rank regulators by predictive importance. Rather than using a stringent threshold, we collected all regulators whose margin-based score is above 0; we reasoned that since the score only measures whether the regulator independently affects the prediction score, a higher threshold could lose more subtle regulators that act cooperatively with other factors. We also note that stabilized boosting retains multiple correlated features in the ADT nodes, which means that all statistically important regulators are identified by the margin score; without stabilization, a regulator might not be included in the tree if it is tightly correlated with another regulator that has been included. For example, if two regulators are perfectly correlated across all experiments, we expect them to occur in exactly the same nodes and therefore give the same margin score.

## Supporting Information

Dataset S1 Normalized gene expression data for all experiments (processed using RMA Express)
(2.76 MB ZIP)

Dataset S2 Discretized gene expression data for all experiments that is used in the MEDUSA learning procedure
(0.23 MB ZIP)

Dataset S3 log_2_ of fold change of gene expression data for all experiments that is used in the MEDUSA learning procedure
(1.29 MB ZIP)

Dataset S4 List of differentially expressed genes (UP - upregulated, DOWN - downregulated) in each of the conditions discussed in the paper
(0.05 MB ZIP)

Dataset S5 Properties of the alternating decision tree learned by MEDUSA. The file lists the regulators and motifs learned by MEDUSA at each iteration. It also lists nodes that precede and follow each node in the ADT.
(0.03 MB ZIP)

Figure S1 Distribution of prediction scores for +1 (red curve), 0 (blue curve), −1 (green curve) examples.
(0.18 MB TIF)

Figure S2 Scatter plot of true expression values versus prediction scores *F*(*x*). The scatter plot shows a high correlation between prediction scores (*y*-axis) and true log expression values (*y*-axis) for all examples. The red, blue, and green points represent the +1, 0, and −1 labeled examples, respectively.
(1.90 MB TIF)

Figure S3 Heat map of target genes that are anaerobically induced in *HAP1* and Δ*hap1* cells, and those that are suppressed by heme. Significant predictive regulators and sequence motifs are also shown.
(1.55 MB TIF)

Figure S4 Heat map of target genes that are anaerobically induced in *HAP1* and Δ*hap1* cells, and those that are induced by Co^2+^ ion. Significant predictive regulators and sequence motifs are also shown.
(1.51 MB TIF)

Figure S5 Heat map of target genes that are anaerobically suppressed in *HAP1* and *Δhap1* cells, and those that are suppressed by Co^2+^ ion. Significant predictive regulators and sequence motifs are also shown.
(1.66 MB TIF)

Figure S6 Conditions used in microarray expression experiments and identified target genes.
(0.59 MB TIF)

Figure S7 Venn diagrams showing the numbers of oxygen-regulated, heme-regulated, and Co^2+^-inducible genes in *HAP1* and *Δhap1* cells. (A) A Venn diagram illustrating the numbers of hypoxically suppressed (oxygen-induced) genes in *HAP1* and *Δhap1* cells, and heme-induced genes. (B) A Venn diagram illustrating the numbers of hypoxically induced (oxygen-suppressed) genes in *HAP1* and *Δhap1* cells, and heme-suppressed genes. (C) A Venn diagram illustrating the numbers of hypoxically induced (oxygen-suppressed) genes in *HAP1* cells at 1.5 or 6 hours after shifting to anaerobic growth conditions. (D) A Venn diagram illustrating the numbers of hypoxically suppressed (oxygen-induced) genes in *HAP1* cells at 1.5 or 6 hours after shifting to anaerobic growth conditions. (E) A Venn diagram illustrating the numbers of hypoxically induced (oxygen-suppressed) genes in *HAP1* and *Δhap1* cells, and Co^2+^-inducible genes.
(1.42 MB TIF)

Figure S8 The functional categorization of identified oxygen-regulated, heme-regulated, and Co^2+^-inducible genes. This figure illustrates the enriched Gene Ontology process annotations for selected sets of the differentially expressed target gene Venn diagrams of Figure S7. Each row represents a set of genes, and each column represents a GO process annotation. The set names are to the left of the color matrix. The GO annotations are shown below the color matrix. Each element of the color matrix illustrates the *p*-value of enrichment. The colorbar on the right shows the colors used for the range of *p*-values. The following naming convention is used for the gene sets. Each set represents the intersection of sets of genes that are differentially expressed in different experimental contexts. Each experimental context is represented by an identifier (e.g., Anaerobic represents the hypoxia condition) followed by up or down arrows denoting up-regulation or down-regulation of the genes in that context. The symbol ‘∼’ is the logical operation ‘NOT’, while the up and down arrows specify whether the set of genes up or downregulated in that condition. For example, Anaerobic↑Anaerobic (Δ*hap1*)↑∼Co↑ refers to the set of target genes that are hypoxically induced both in *HAP1* and Δ*hap1* cells but are not Co^2+^-inducible. The *p*-values shown are not corrected for multiple comparisons.
(1.46 MB TIF)

Figure S9 Comparison of MacIsaac et al. PSSMs to PSSMs learned by MEDUSA. MacIsaac et al. used ChIP-chip data to identify potential binding sites for 124 transcription factors. We use the symmetrized Kullback-Leibler (KL) distance to identify the best matching MEDUSA PSSM to each of these 124 PSSMs. The transcription factors are listed in ascending order by the KL distance to the best match. Column 3 shows the MacIsaac et al. PSSMs and Column 4 shows the best matching MEDUSA PSSM.
(1.99 MB TIF)

Figure S10 Positive tail of the empirical null distribution for normalized margin scores. The figure shows the normalized histogram for positive margin scores (margin score >0) for all gene sets in all the randomization trials. We normalize by dividing the frequency in each bin by the total number of data points (|margin scores >0|+|margin scores ≤0|). The highest observed margin score computed from the randomized data was 0.53. The red points are the 54 regulators with positive margin score using the true labels; if a regulator was identified for multiple gene sets, its most significant *p*-value is shown. We see that most of these points lie far into the positive tail of the distribution.
(10.19 MB TIF)

Figure S11 Empirical *p*-values for the (normalized) margin score. We calculate a *p*-value for each margin score θ as the fraction of data points in the randomization trials with margin score >θ. The 54 regulators with positive margin scores using true labels have low *p*-values. If a regulator was identified for multiple gene sets, its most significant *p*-value is shown.
(10.19 MB TIF)

Figure S12 Estimated false discovery rate for choices of *p*-value threshold for 54 identified regulators. We obtain *p*-values for all 507 regulators in each of the 12 gene sets. We then apply the step-wise Benjamini-Hochberg procedure to this set of *p*-values to obtain the FDR corresponding to each *p*-value cutoff. The figure shows the best margin score, corresponding *p*-value and FDR for the 54 regulators with positive margin score in at least one gene set. The top 16 regulators correspond to very small FDRs and include many known hypoxia regulators such as Upc2, Hap4, Mga2, Rox1, and Hap1, as well as novel regulators.
(1.34 MB TIF)

Text S1 Supplemental methods. This file contains additional supplemental information such as additional context-specific results and detailed cross-validation and prediction accuracy results and comparisons.
(3.97 MB DOC)
