# Supplementary figures and images for: A Predictive Model of the Oxygen and Heme Regulatory Network in Yeast
Source: PLoS Comput Biol. 2008 Nov 14;4(11):e1000224. doi: 10.1371/journal.pcbi.1000224 (PMC2573020; doi:10.1371/journal.pcbi.1000224)

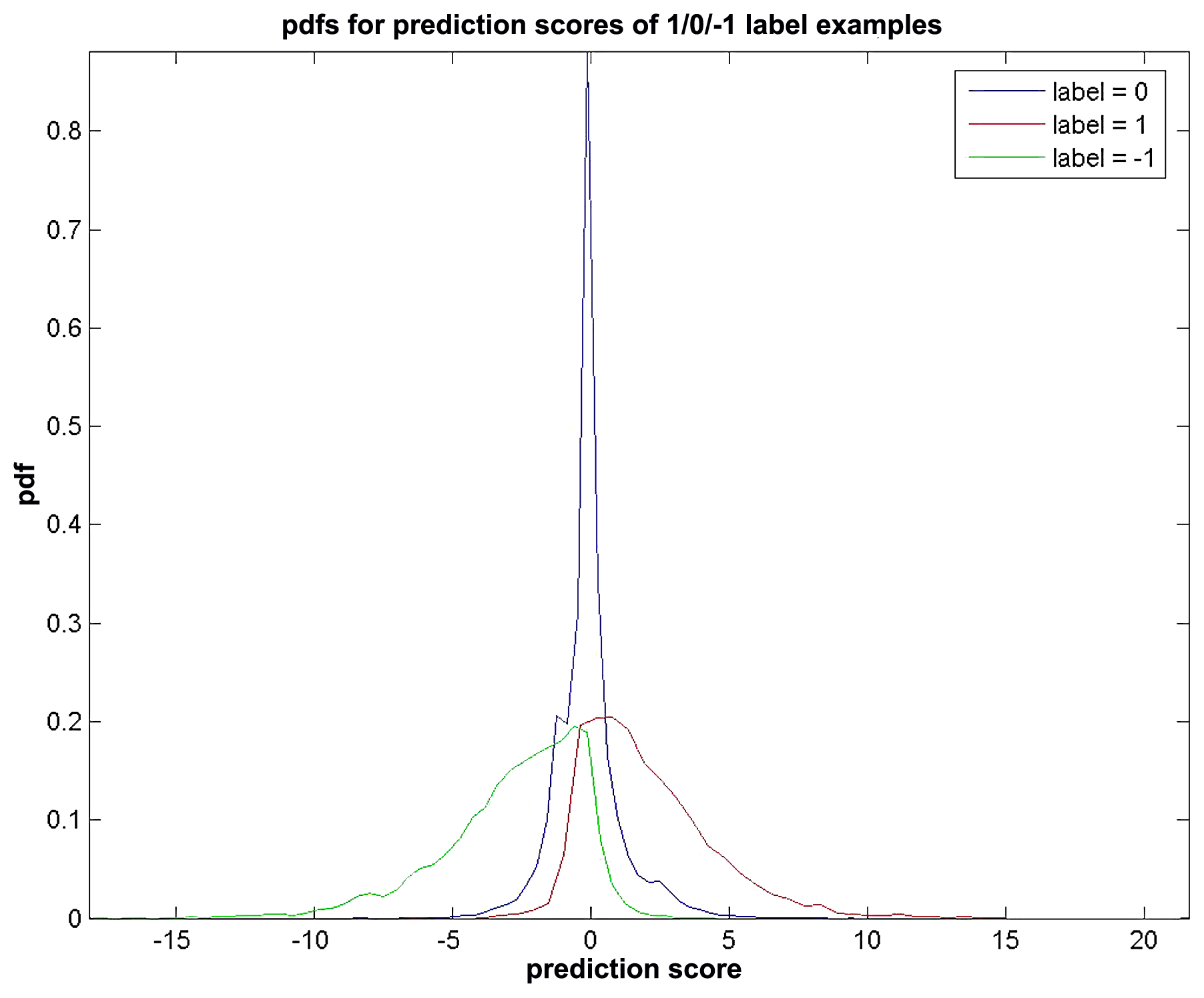

Supplement: Figure S1 — Distribution of prediction scores for +1 (red curve), 0 (blue curve), −1 (green curve) examples. (0.18 MB TIF) [file pcbi.1000224.s006.tif]

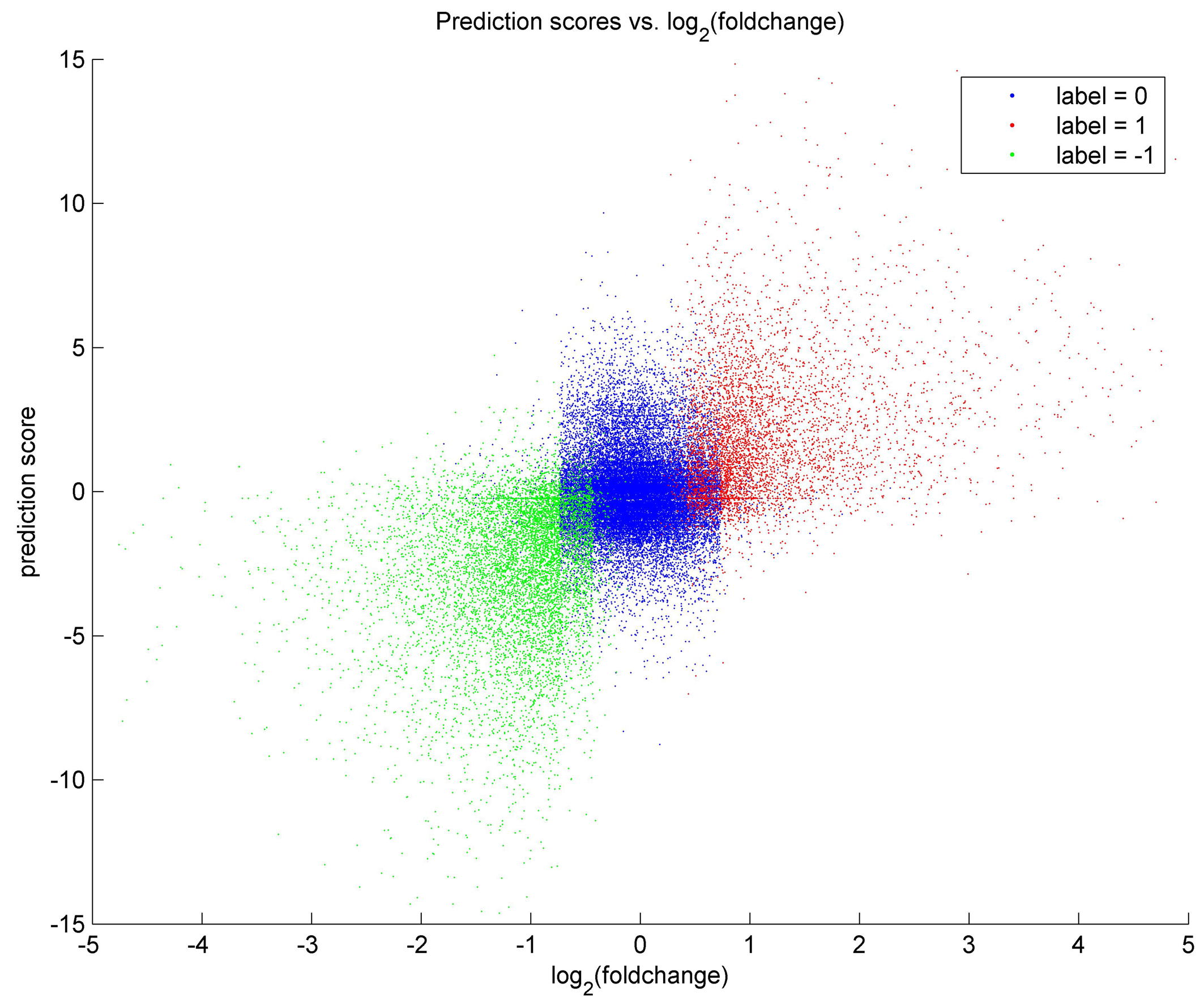

Supplement: Figure S2 — Scatter plot of true expression values versus prediction scores F(x). The scatter plot shows a high correlation between prediction scores (y-axis) and true log expression values (y-axis) for all examples. The red, blue, and green points represent the +1, 0, and −1 labeled examples, respectively. (1.90 MB TIF) [file pcbi.1000224.s007.tif]

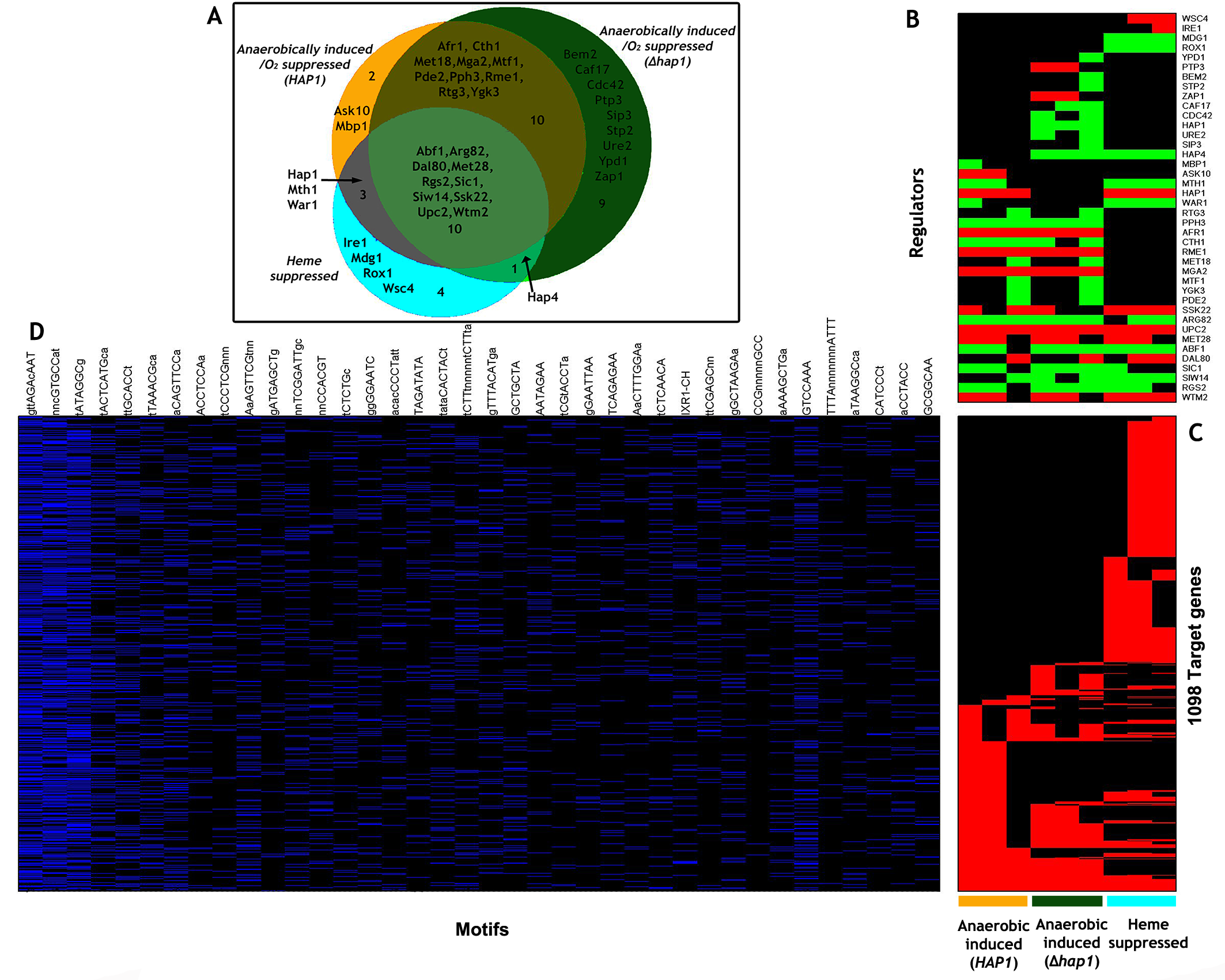

Supplement: Figure S3 — Heat map of target genes that are anaerobically induced in HAP1 and Δhap1 cells, and those that are suppressed by heme. Significant predictive regulators and sequence motifs are also shown. (1.55 MB TIF) [file pcbi.1000224.s008.tif]

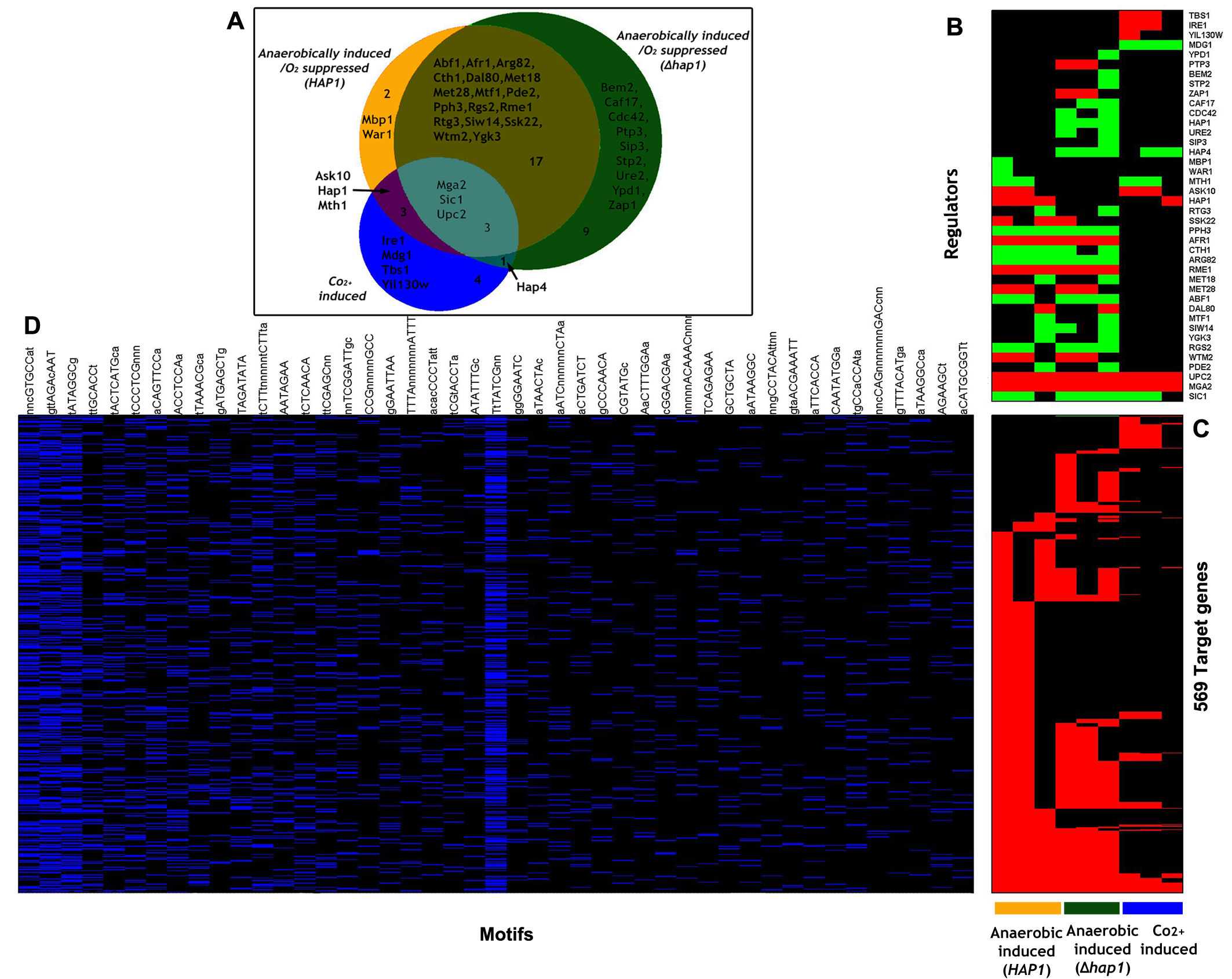

Supplement: Figure S4 — Heat map of target genes that are anaerobically induced in HAP1 and Δhap1 cells, and those that are induced by Co2+ ion. Significant predictive regulators and sequence motifs are also shown. (1.51 MB TIF) [file pcbi.1000224.s009.tif]

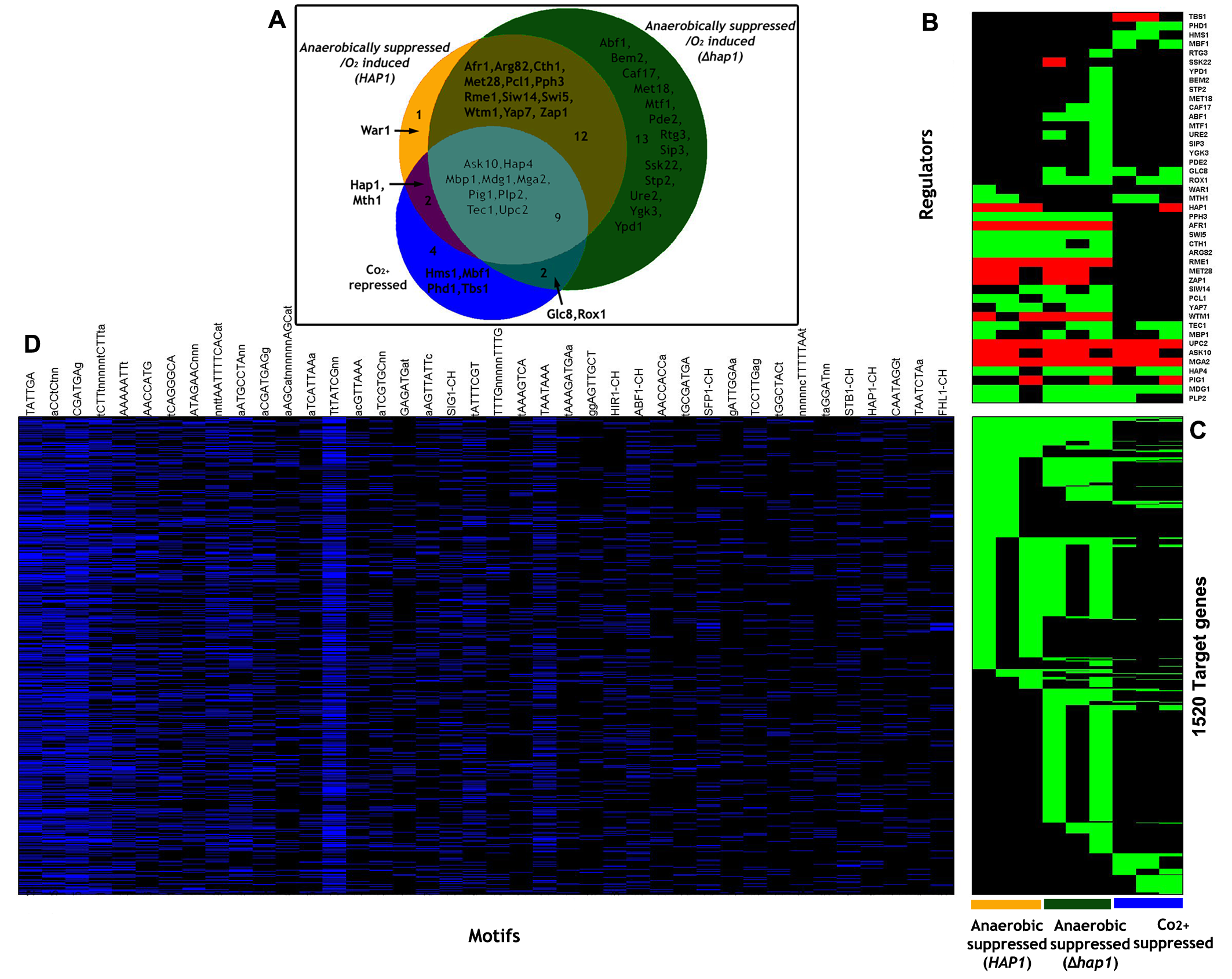

Supplement: Figure S5 — Heat map of target genes that are anaerobically suppressed in HAP1 and Δhap1 cells, and those that are suppressed by Co2+ ion. Significant predictive regulators and sequence motifs are also shown. (1.66 MB TIF) [file pcbi.1000224.s010.tif]

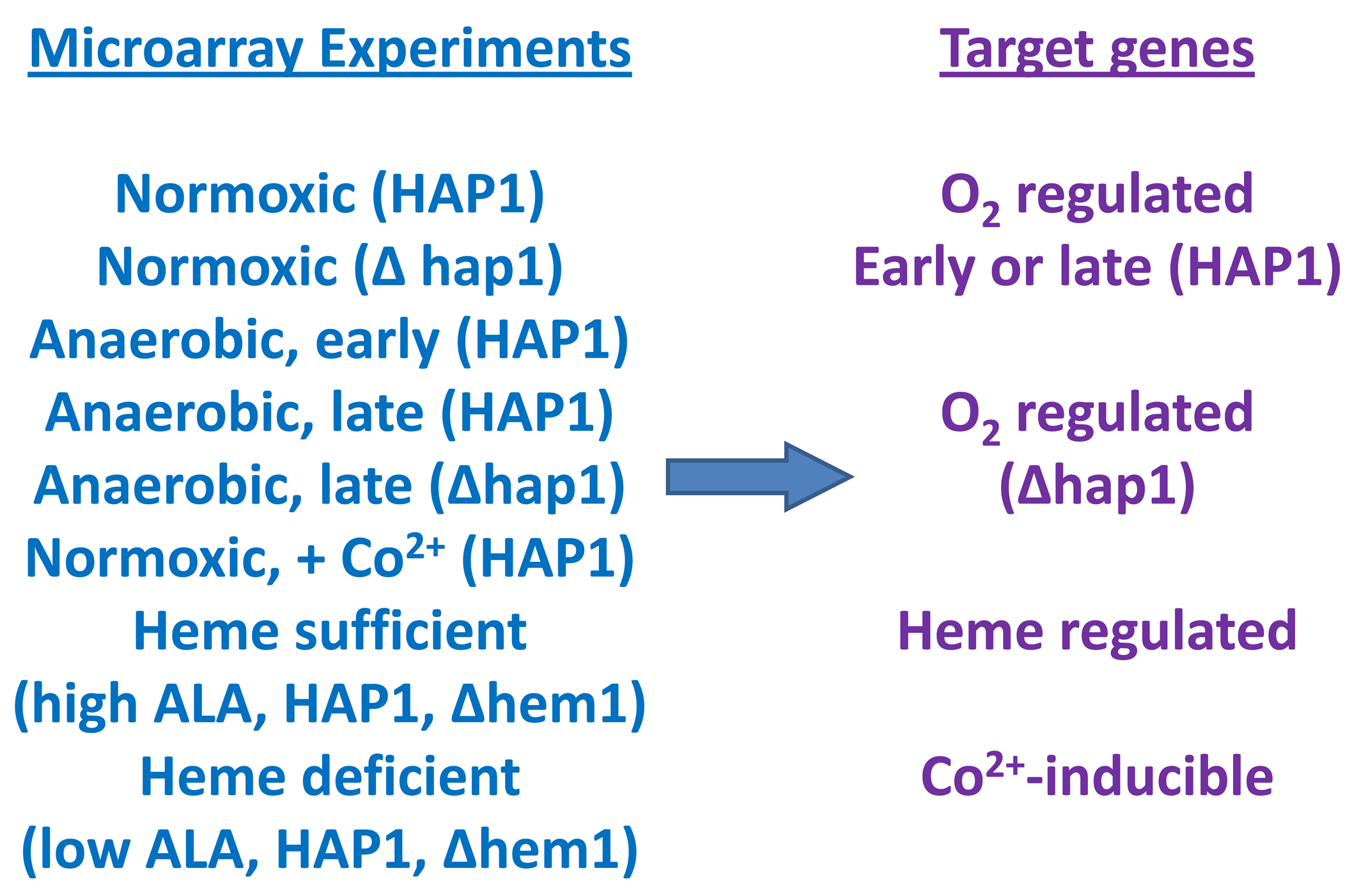

Supplement: Figure S6 — Conditions used in microarray expression experiments and identified target genes. (0.59 MB TIF) [file pcbi.1000224.s011.tif]

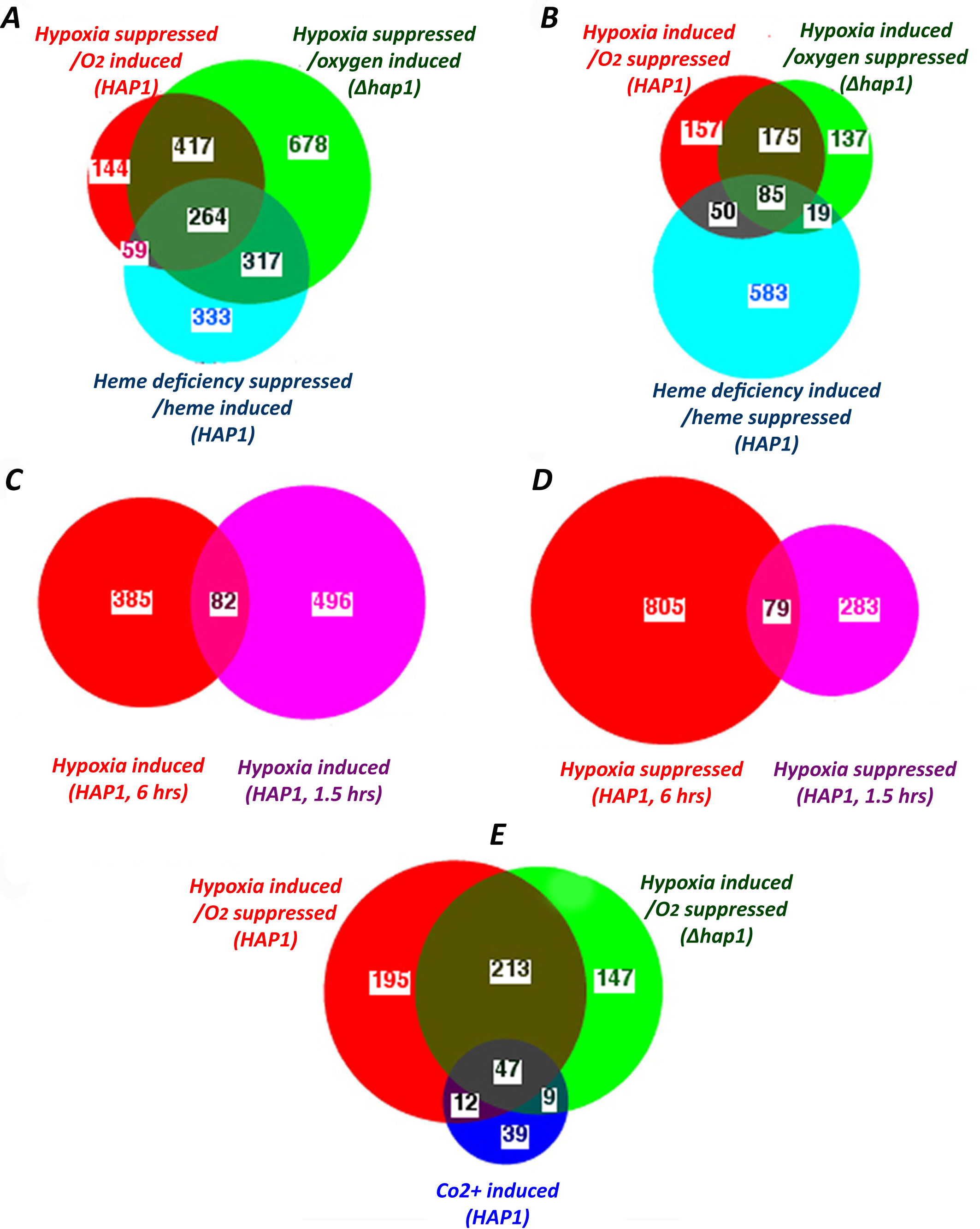

Supplement: Figure S7 — Venn diagrams showing the numbers of oxygen-regulated, heme-regulated, and Co2+-inducible genes in HAP1 and Δhap1 cells. (A) A Venn diagram illustrating the numbers of hypoxically suppressed (oxygen-induced) genes in HAP1 and Δhap1 cells, and heme-induced genes. (B) A Venn diagram illustrating the numbers of hypoxically induced (oxygen-suppressed) genes in HAP1 and Δhap1 cells, and heme-suppressed genes. (C) A Venn diagram illustrating the numbers of hypoxically induced (oxygen-suppressed) genes in HAP1 cells at 1.5 or 6 hours after shifting to anaerobic growth conditions. (D) A Venn diagram illustrating the numbers of hypoxically suppressed (oxygen-induced) genes in HAP1 cells at 1.5 or 6 hours after shifting to anaerobic growth conditions. (E) A Venn diagram illustrating the numbers of hypoxically induced (oxygen-suppressed) genes in HAP1 and Δhap1 cells, and Co2+-inducible genes. (1.42 MB TIF) [file pcbi.1000224.s012.tif]

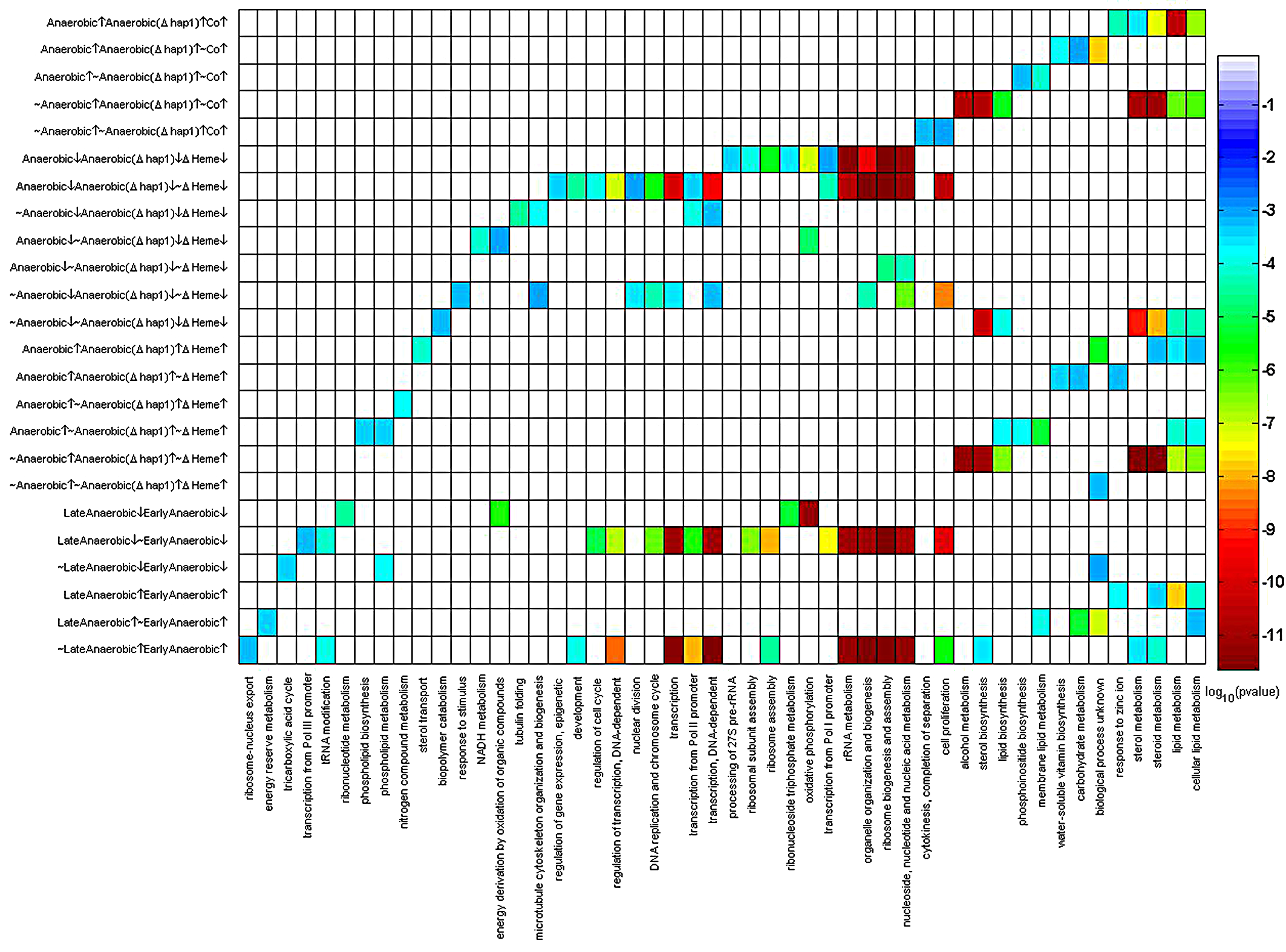

Supplement: Figure S8 — The functional categorization of identified oxygen-regulated, heme-regulated, and Co2+-inducible genes. This figure illustrates the enriched Gene Ontology process annotations for selected sets of the differentially expressed target gene Venn diagrams of Figure S7. Each row represents a set of genes, and each column represents a GO process annotation. The set names are to the left of the color matrix. The GO annotations are shown below the color matrix. Each element of the color matrix illustrates the p-value of enrichment. The colorbar on the right shows the colors used for the range of p-values. The following naming convention is used for the gene sets. Each set represents the intersection of sets of genes that are differentially expressed in different experimental contexts. Each experimental context is represented by an identifier (e.g., Anaerobic represents the hypoxia condition) followed by up or down arrows denoting up-regulation or down-regulation of the genes in that context. The symbol ‘∼’ is the logical operation ‘NOT’, while the up and down arrows specify whether the set of genes up or downregulated in that condition. For example, Anaerobic↑Anaerobic (Δhap1)↑∼Co↑ refers to the set of target genes that are hypoxically induced both in HAP1 and Δhap1 cells but are not Co2+-inducible. The p-values shown are not corrected for multiple comparisons. (1.46 MB TIF) [file pcbi.1000224.s013.tif]

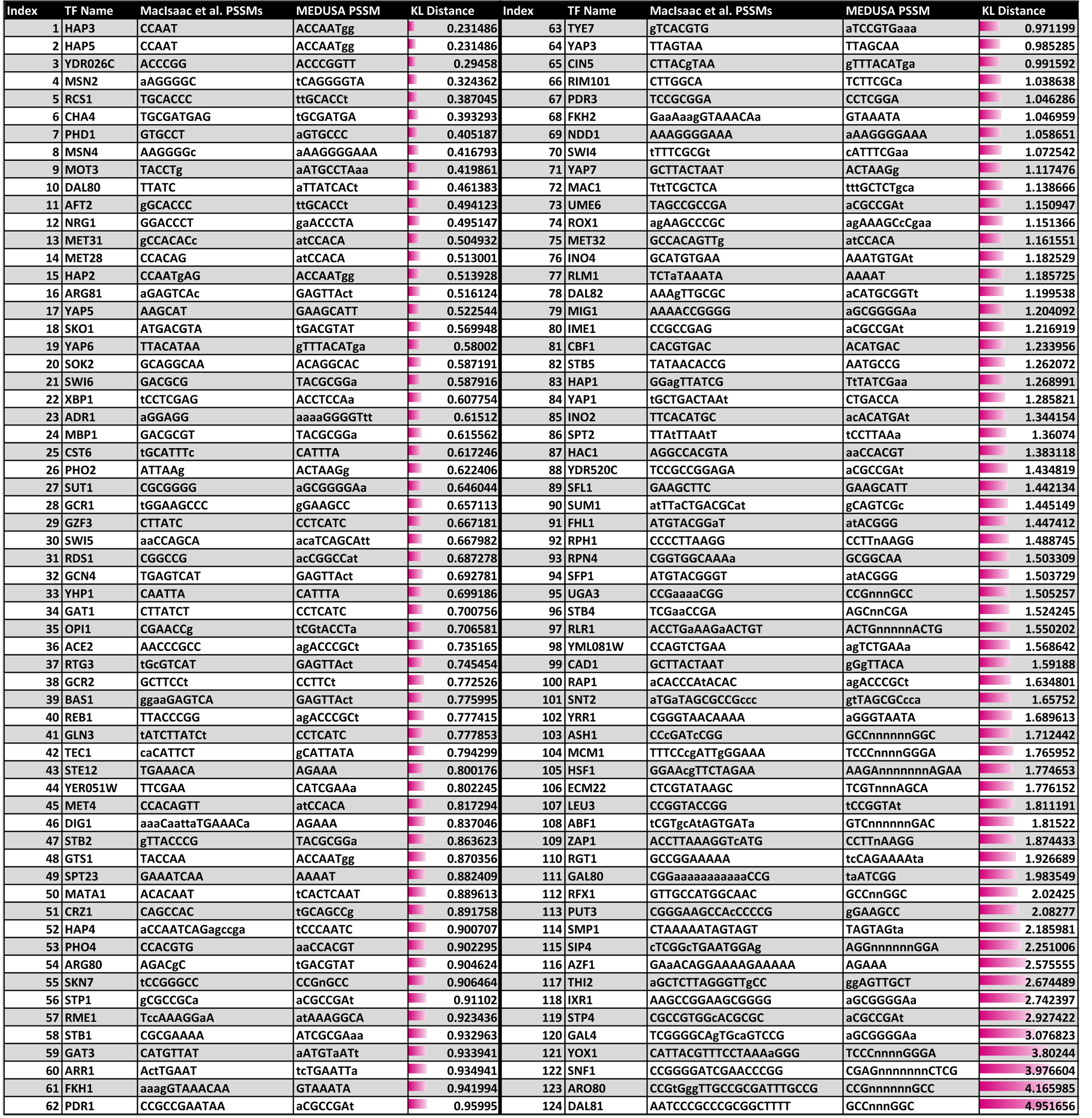

Supplement: Figure S9 — Comparison of MacIsaac et al. PSSMs to PSSMs learned by MEDUSA. MacIsaac et al. used ChIP-chip data to identify potential binding sites for 124 transcription factors. We use the symmetrized Kullback-Leibler (KL) distance to identify the best matching MEDUSA PSSM to each of these 124 PSSMs. The transcription factors are listed in ascending order by the KL distance to the best match. Column 3 shows the MacIsaac et al. PSSMs and Column 4 shows the best matching MEDUSA PSSM. (1.99 MB TIF) [file pcbi.1000224.s014.tif]

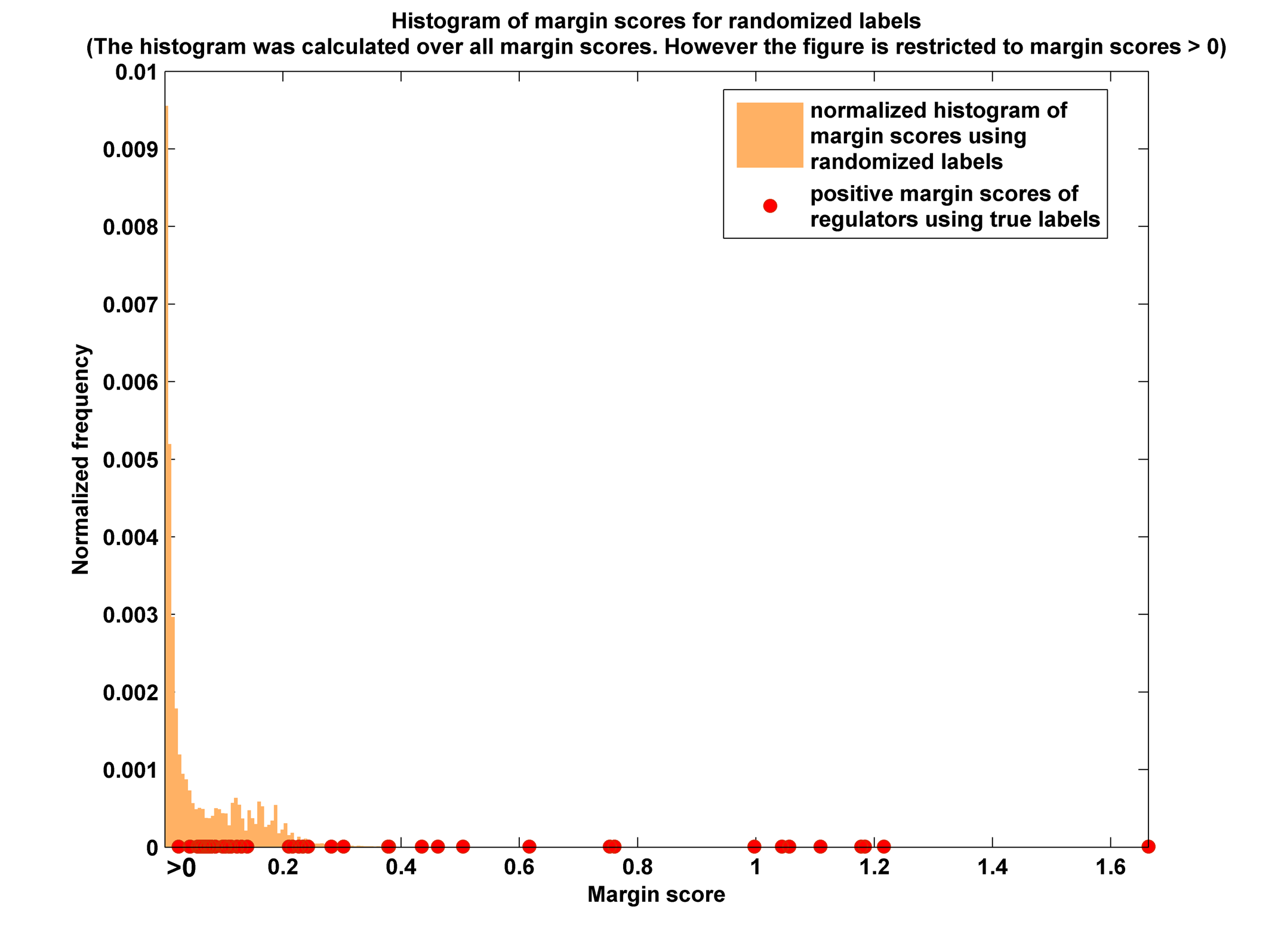

Supplement: Figure S10 — Positive tail of the empirical null distribution for normalized margin scores. The figure shows the normalized histogram for positive margin scores (margin score >0) for all gene sets in all the randomization trials. We normalize by dividing the frequency in each bin by the total number of data points (|margin scores >0|+|margin scores ≤0|). The highest observed margin score computed from the randomized data was 0.53. The red points are the 54 regulators with positive margin score using the true labels; if a regulator was identified for multiple gene sets, its most significant p-value is shown. We see that most of these points lie far into the positive tail of the distribution. (10.19 MB TIF) [file pcbi.1000224.s015.tif]

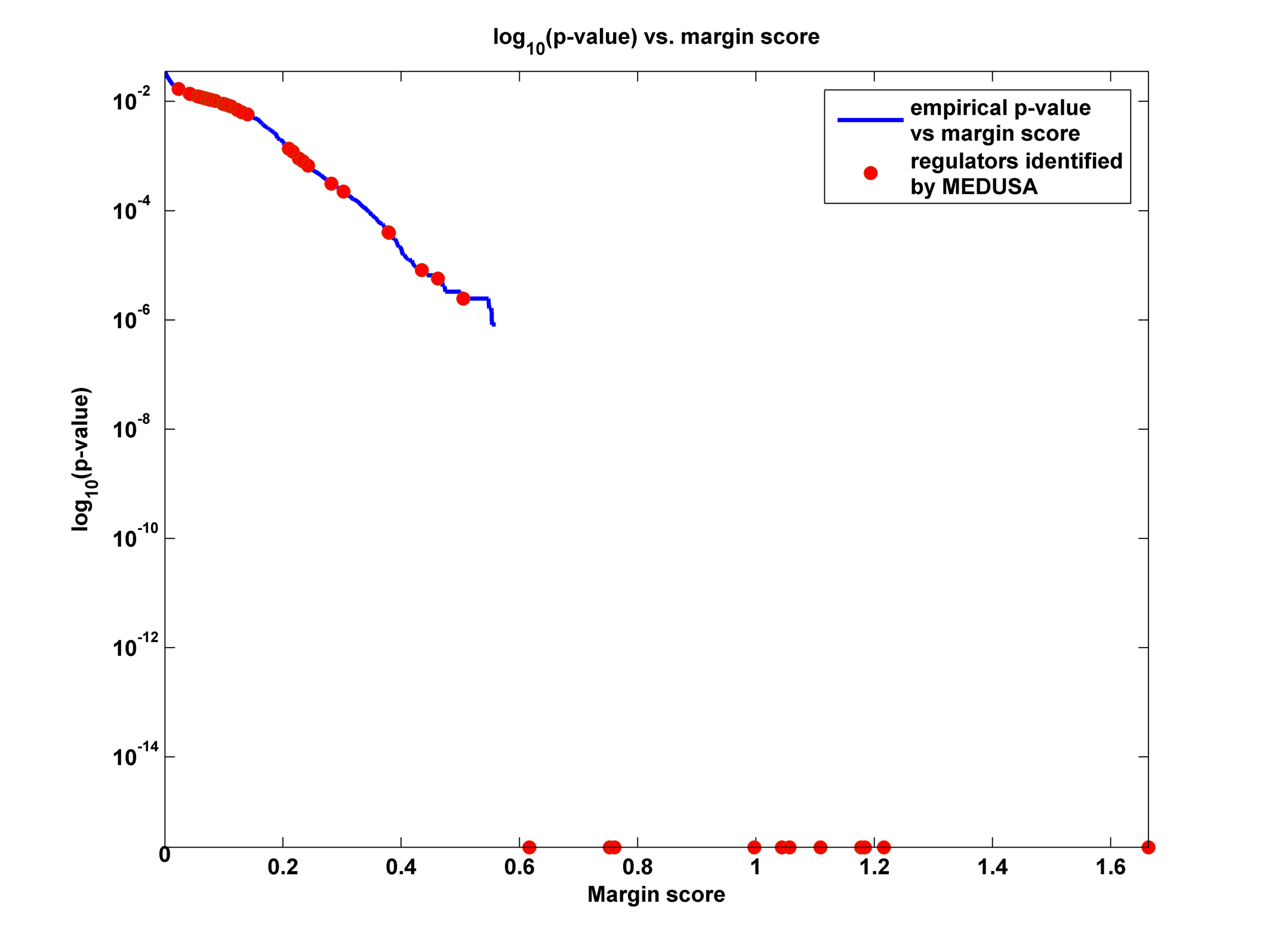

Supplement: Figure S11 — Empirical p-values for the (normalized) margin score. We calculate a p-value for each margin score θ as the fraction of data points in the randomization trials with margin score >θ. The 54 regulators with positive margin scores using true labels have low p-values. If a regulator was identified for multiple gene sets, its most significant p-value is shown. (10.19 MB TIF) [file pcbi.1000224.s016.tif]

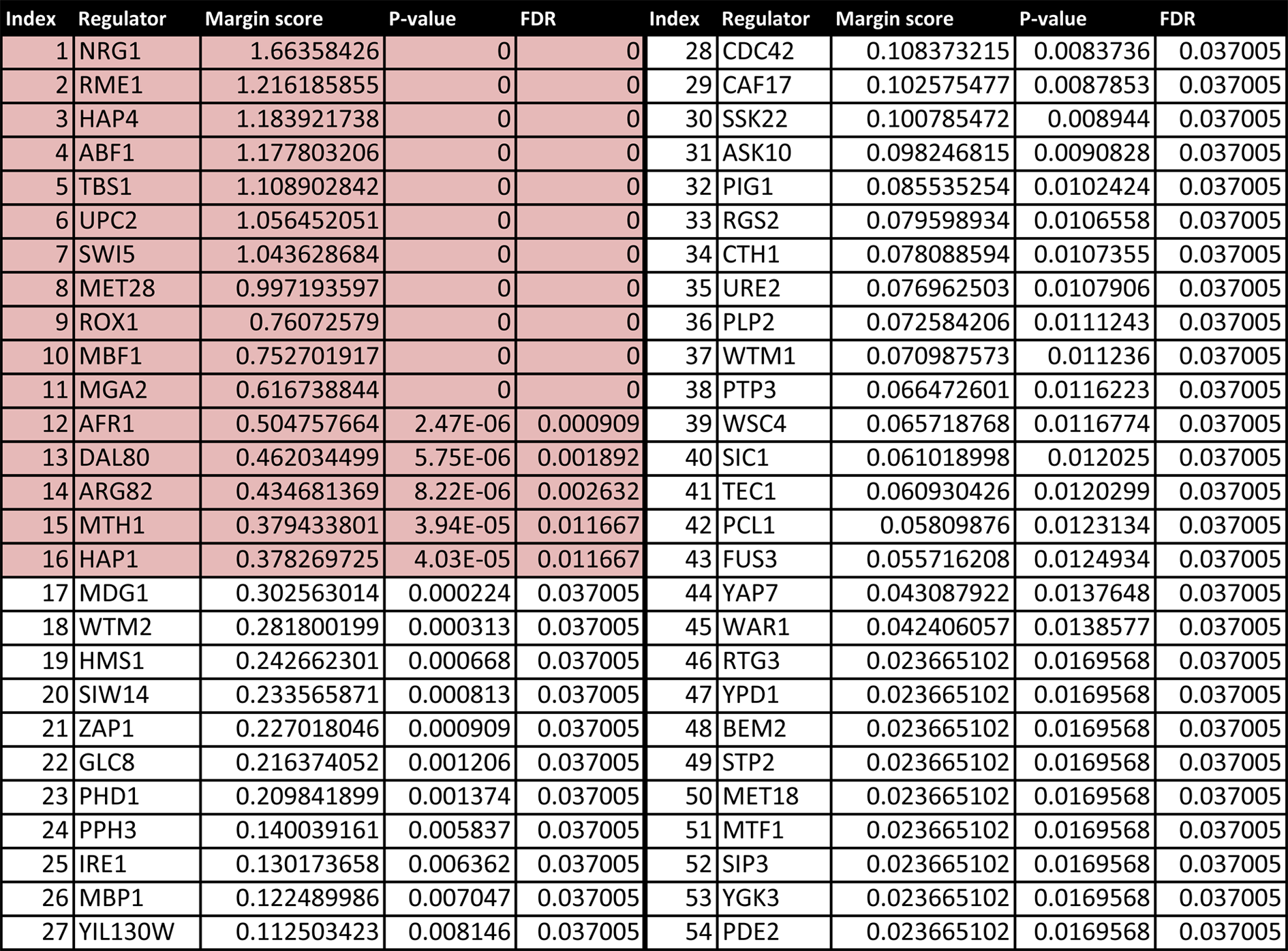

Supplement: Figure S12 — Estimated false discovery rate for choices of p-value threshold for 54 identified regulators. We obtain p-values for all 507 regulators in each of the 12 gene sets. We then apply the step-wise Benjamini-Hochberg procedure to this set of p-values to obtain the FDR corresponding to each p-value cutoff. The figure shows the best margin score, corresponding p-value and FDR for the 54 regulators with positive margin score in at least one gene set. The top 16 regulators correspond to very small FDRs and include many known hypoxia regulators such as Upc2, Hap4, Mga2, Rox1, and Hap1, as well as novel regulators. (1.34 MB TIF) [file pcbi.1000224.s017.tif]
