# Supplementary material for: A Predictive Model of the Oxygen and Heme Regulatory Network in Yeast
Source: PLoS Comput Biol. 2008 Nov 14;4(11):e1000224. doi: 10.1371/journal.pcbi.1000224 (PMC2573020; doi:10.1371/journal.pcbi.1000224)
Supplement: Text S1 — Supplemental methods. This file contains additional supplemental information such as additional context-specific results and detailed cross-validation and prediction accuracy results and comparisons. (3.97 MB DOC) [file pcbi.1000224.s018.doc]

**Supplemental Methods**

A Predictive Model of the Oxygen Sensing and Regulatory Network in Yeast

Anshul Kundaje^1^, Xiantong Xin^2^, Changgui Lan^2^, Steve Lianoglou^3,4^, Mei Zhou^2^,

Li Zhang*^2^, Christina Leslie*^4^

^1^Department of Computer Science

Columbia University

1214 Amsterdam Ave, MC 0401, New York, NY 10027

^2^Department of Molecular and Cell Biology

University of Texas at Dallas, Mail Stop FO31

800 W. Campbell Road, Richardson, TX 75080

^3^Department of Physiology, Biophysics, and Systems Biology

Weill Medical College of Cornell University

1300 York Avenue, New York, NY 10065

^4^Computational Biology Program

Memorial Sloan-Kettering Cancer Center

1275 York Ave, Mail Box #460, New York, NY 10065

*Corresponding author contact information:

Phone: (646)-888-2762 Fax: (646)-422-0717 (CL)

Email: [lz2115@columbia.edu](mailto:lz2115@columbia.edu) (LZ), [cleslie@cbio.mskcc.org](mailto:cleslie@cs.columbia.edu) (CL)

*Analysis of expression signatures identifies several broad functional categories*

We performed GO functional analysis on the 16 expression signatures identified by perturbation of the oxygen regulatory network (Figure 1 in the main text) and found significant enrichment of functional terms for most signatures.

Signature 1 (sig1) consists of 70 genes that are strongly upregulated 6 hours into anaerobosis independent of Hap1 deletion. They are also upregulated in response to heme deletion, as opposed to the 267 genes in signature 3 (sig3) which are exclusively upregulated in the late hypoxia experiments. Sig1 genes are mainly involved in cell wall biogenesis (5.9e-07) and stress response (2.6e-06). The biotin biosynthesis gene cassette (BIO3/4/5) is also part of this group. Sig3 on the other hand is enriched for genes involved in carbohydrate and alcohol metabolism (2.4e-07). Signature 15 (sig15) shows strong induction in late hypoxia similar to sig3. However, sig15 genes are also significantly downregulated in early hypoxia. This set is also weakly enriched for genes involved in carbohydrate metabolism (1.4e-05). Signature 2 (sig2) is enriched for several essential transcription factors (1e-14) and rRNA/ribosome processing genes (1e-14). These 207 genes are induced in early hypoxia but suppressed at the 6 hour time point independent of Hap1 deletion. These genes were also significantly downregulated in the heme deletion experiment. Signature 5 (sig5) is diametrically opposite to sig1 but functionally similar to sig2. It consists of typical stress suppressed genes involved in ribosome biogenesis (2.5e-10).

Signature 6 (sig6) consists of 160 genes several of which are involved in ATP synthesis dependent proton transport and respiration (3.1e-10). These genes are strongly suppressed in all but the *∆hap1* condition, indicating that they might not be regulated by Hap1. However, signature 16 (sig16) consists of 34 Hap1*-*dependent genes that are strongly suppressed in all conditions including the *∆hap1* experiment. These genes (such as the COX and QCR genes) are involved in aerobic respiratory processes (1.0e-14) , electron transport (4.4e-14) and heme-dependent oxidoreducatase activity (5.8e-14). Signature 8 (sig8) shows strong downregulation in *∆heme* and late hypoxia (*∆hap1)* experiments and weak suppression in the other conditions. It is mainly made up of structural constituents of ribosomes (1.4e-06) and other genes involved in protein synthesis and metabolism (5.8e-06).

Signature 9 (sig9) and 10 (sig10) consist of genes significantly downregulated in the late hypoxia conditions. However, sig9 also shows weak induction in early hypoxia. Sig9 is made up of several cell cycle genes (5.6e-12). The histone genes (3.0e-07) are part of sig10. Genes in signature 11 (sig11) and signature 14 (sig14) appear to be strongly regulated by heme. Sig11 genes are exclusively induced by heme deletion whereas sig14 genes are suppressed in the same experiment. The ergosterol biosynthesis genes which are part of sig14 are known to be heme regulated. Sig11 is an intriguing set of genes made up of 500 genes of which 354 are functionally uncharacterized (3.2e-13). These could be an important class of heme-regulated genes. Signature 13 (sig13) consists of 276 genes many of which are transcription factors (3.15e-14) and signal transduction factors (7.23e-08). These genes are exclusively induced in early hypoxia and seem to represent an early regulatory response.

MEDUSA achieves high prediction accuracy for the three-class (up/down/baseline) prediction problem

MEDUSA uses only the up/down-regulated examples for training and testing, constituting 12% of the gene expression data in our study. However, it is possible to make three-class predictions (up, down, or baseline) by thresholding MEDUSA’s prediction scores, and in this way we can report 3-class cross-validation accuracy across all examples, including those that are labeled baseline.

The output of the learning algorithm is a real-valued prediction function $F(g,e)$ for all genes and experiments in the form of an alternating decision tree. The sign of the prediction score gives the predicted label and the absolute value represents a confidence level for the prediction. We can make 3-class predictions by thresholding on the confidence levels of up and down predictions, that is, we predict examples to be up- or down-regulated if $F(g,e)>a$ or $F(g,e)<-b$, and to be baseline if $-b<=F(g,e)<=a$ where $a,b>0$.

We reexamined our 10-fold cross-validation results using held-out examples to evaluate three-class prediction performance, where baseline examples were randomly divided among the 10 folds for the purpose of reporting results. Figure S1 shows the distribution of the prediction scores for the three classes, and Figure S2 shows a scatterplot of the true log_2_ expression values versus prediction scores for all examples. In both figures, we see a good separation between classes, and in the scatterplot, we see a significant correlation between true expression level and real-valued prediction score.

| 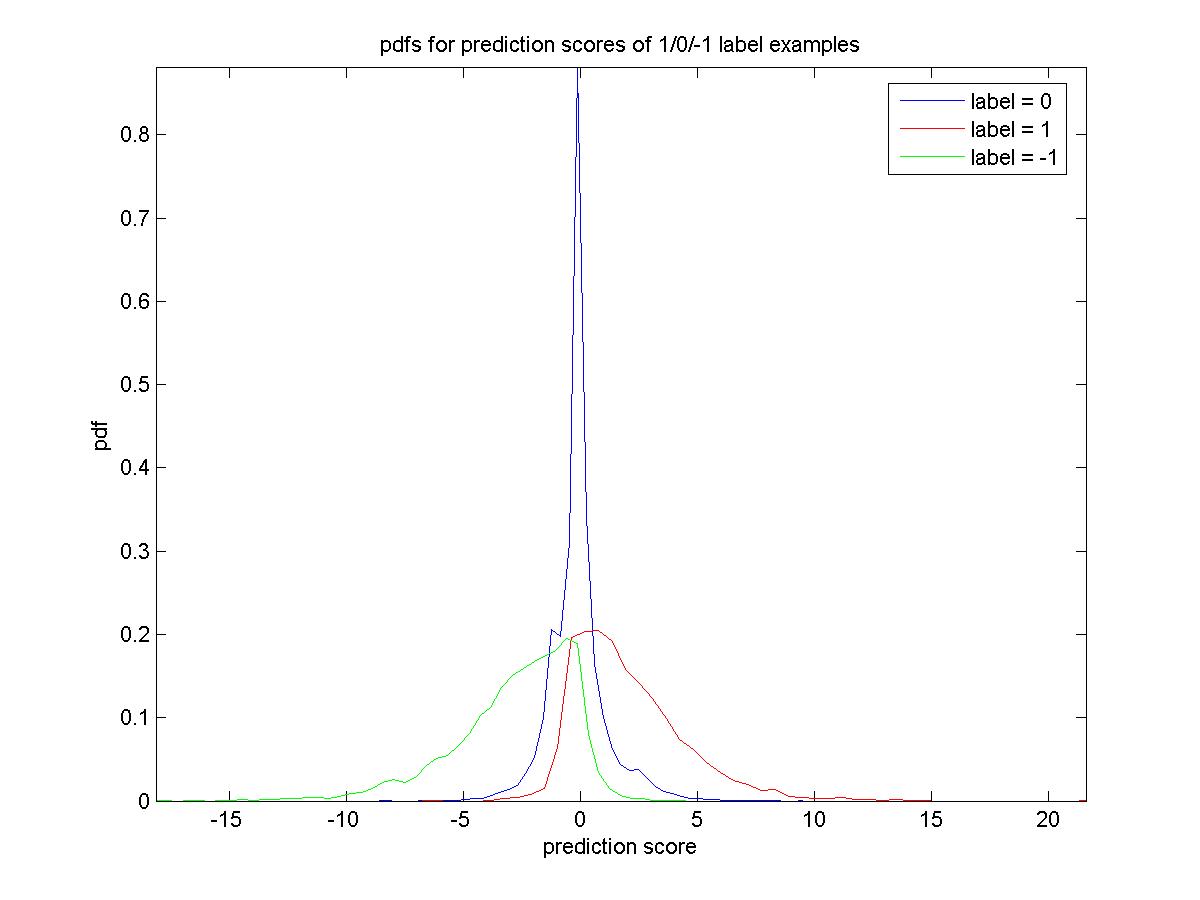Figure S1. Distribution of prediction scores for +1 (red curve), 0 (blue curve), -1 (green curve) examples. |
| --- |
|  |

| Figure S2. Scatter plot of true expression values versus prediction scores F(x). The scatter plot shows a high correlation between prediction scores (y-axis) and true log expression values (y-axis) for all examples. The red, blue and green points represent the +1, 0 and -1 labeled examples respectively.  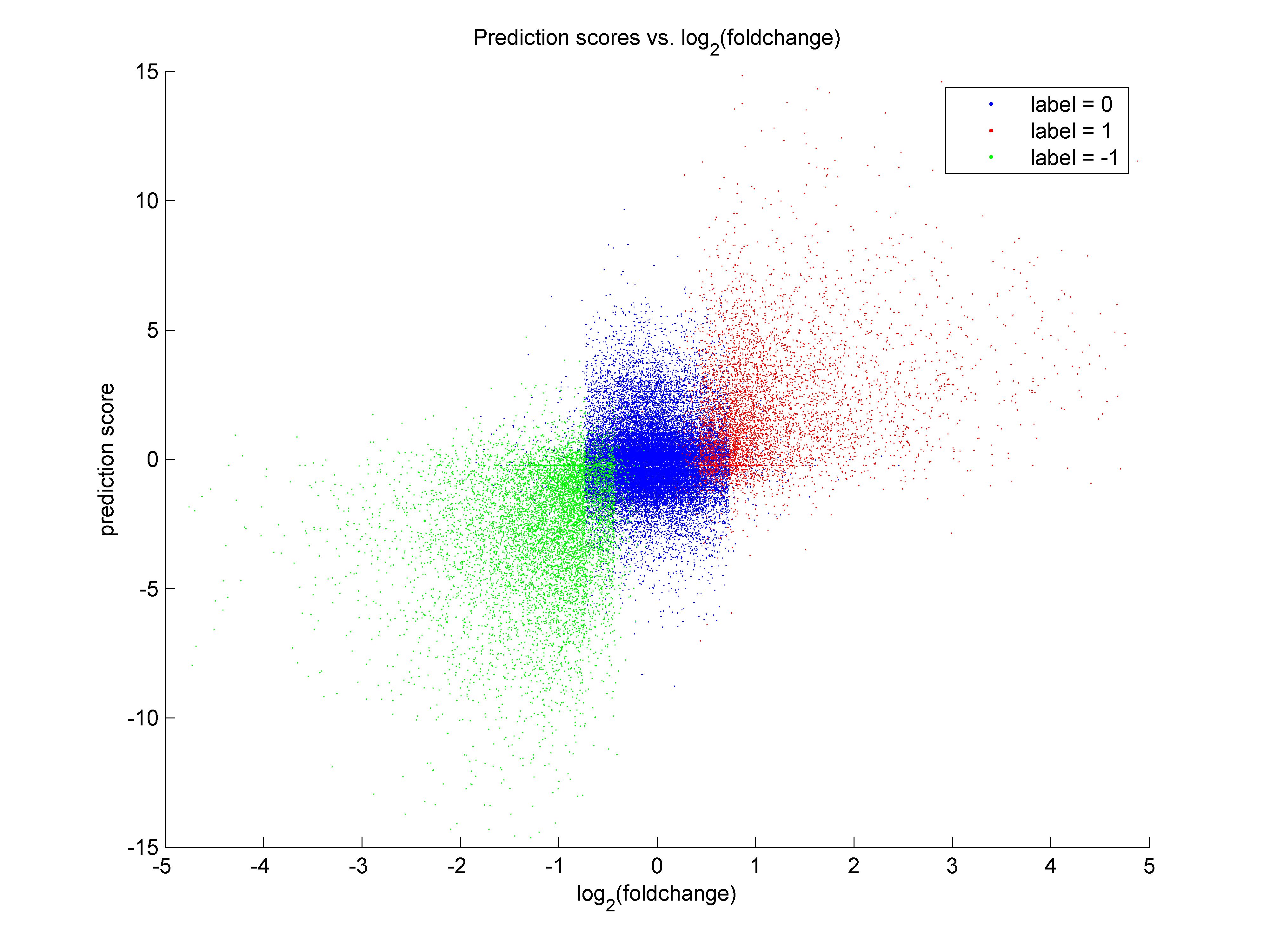 |
| --- |
|  |

We chose the confidence thresholds $a$and $-b$ so as to optimize the mean balanced accuracy (average accuracy over the three classes) over the 10 folds. We found the minimum balanced accuracy to be 70.1% for $a=0.49$ and$-b=-1.21$. Performance of a random classifier would yield a balanced accuracy of 33.3%. While it would be more correct to choose the thresholds based on a separate cross-validation scheme, we observed that the balanced error was fairly stable across folds, suggesting that our choice would not lead to overrated performance. For these thresholds, we obtained the confusion matrix shown in Table 1, demonstrating strong diagonal entries and reasonable accuracy on the baseline examples, despite the fact that these examples were omitted from training.

Supplementary Table 1. Truth and predictions for all genes in the held-out experiments, including those expressed at baseline levels. Examples are binned by assigning thresholds a = 0.49 and –b = -1.21 for prediction of positive and negative labels, respectively.

|  | | | Predicted labels |  |
| --- | --- | --- | --- | --- |
| True label |  | +1 | 0 | -1 |
|  | +1 | 71.6% | 26.5% | 2.0% |
|  | 0 | 18.3% | 69.9% | 11.8% |
|  | -1 | 3.3% | 27.8% | 68.9% |

Supplementary Table 2. Dependence of results on sequence features

| Sequence features provided to MEDUSA | 10-fold c.v. accuracy  (450 boosting iterations) |
| --- | --- |
| 500 bp promoters + ChIP chip data | 92% |
| 1000 bp promoters + ChIP chip data | 92% |
| ChIP chip data only | 74% |

*Properties of the Alternating Decision Tree learned by MEDUSA*

We ran MEDUSA for 440 iterations. The resulting alternating decision tree (ADT) is shown in additional file 8. The tree consists of 440 nodes. Each node consists of a set of regulators in combination with a set of PSSMs and/or ChIP chip occupancy profiles. The PSSMs learned by MEDUSA dominate the tree. The earliest ChIP chip feature is observed at iteration 121. It is the ChIP chip occupancy profile of the Hap1 transcription factor. The ADT is at most 3 levels deep. There are 351 single node paths, 87 paths consisting of 2 nodes and only 2 paths consisting of 3 nodes.

*MEDUSA achieves high prediction accuracy when using different promoter sequence lengths but lower accuracy when using ChIP chip data alone*

To evaluate the dependence of our results on promoter sequence length, we tested the MEDUSA using promoter sequences of length 500 bp and 1000 bp in 10-fold cross validation experiments on randomly held out examples, and we found no significance in accuracy. However, when we reran the MEDUSA experiments using only ChIP chip transcription factor occupancies as sequence features, accuracy was significantly poorer. These results indicate that the sequence motif features discovered by MEDUSA are more useful for predicting target gene expression than ChIP chip data. All prediction accuracies were reported for target genes excluding the 503 regulators, which are also used as training examples. Evaluation of the accuracies over all target genes including regulators did not change the results.

*MEDUSA identifies significant condition-specific regulators for induced and suppressed target genes in multiple conditions*

Figures S3, S4 and S5 illustrate the statistically significant regulators for sets of target genes under different conditions of study. Figure S3 presents target genes that are anaerobically induced in *HAP1* and *Δhap1* cells, and those that are suppressed by heme. Figure S4 shows regulation of target genes that are anaerobically induced in *HAP1* and *Δhap1* cells, and those that are induced by Cobalt. Figure S5 shows regulation of target genes that are anaerobically suppressed in *HAP1* and *Δhap1* cells, and those that are suppressed by Cobalt.

In each of the figures, Part (A) shows Venn diagrams illustrating the regulators involved in controlling target genes in the three experimental conditions shown in the figure. For each experiment, the statistically significant regulators associated with the set of target genes are determined by use of a margin-based score (see Methods). Part (B) shows patterns of up (red), down (green), and baseline (black) expression levels for the statistically significant regulators controlling target genes across the three experimental conditions. At the left of each row, the number of target genes affected by the regulator in these experiments is given. Part (C) illustrates the top-ranked sequence features learned by MEDUSA, as determined by a margin-based score, and their hits across the set of target gene promoters. The PSSMs learned by MEDUSA are represented by their consensus patterns. ChIP-chip occupancy features also occur in the list of most significant features. For example in Figure 3, HAP1-CH refers to ChIP chip occupancy by the transcription factor Hap1. At the bottom of each column, the number of target genes containing the motif or feature is given. Part (D) of the figure shows discretized gene expression levels for the differentially regulated target gene list from each of the three experimental conditions.

**Figure S3.** Heat map of target genes that are anaerobically induced in *HAP1* and *Δhap1* cells, and those that are suppressed by heme. Significant predictive regulators and sequence motifs are also shown.


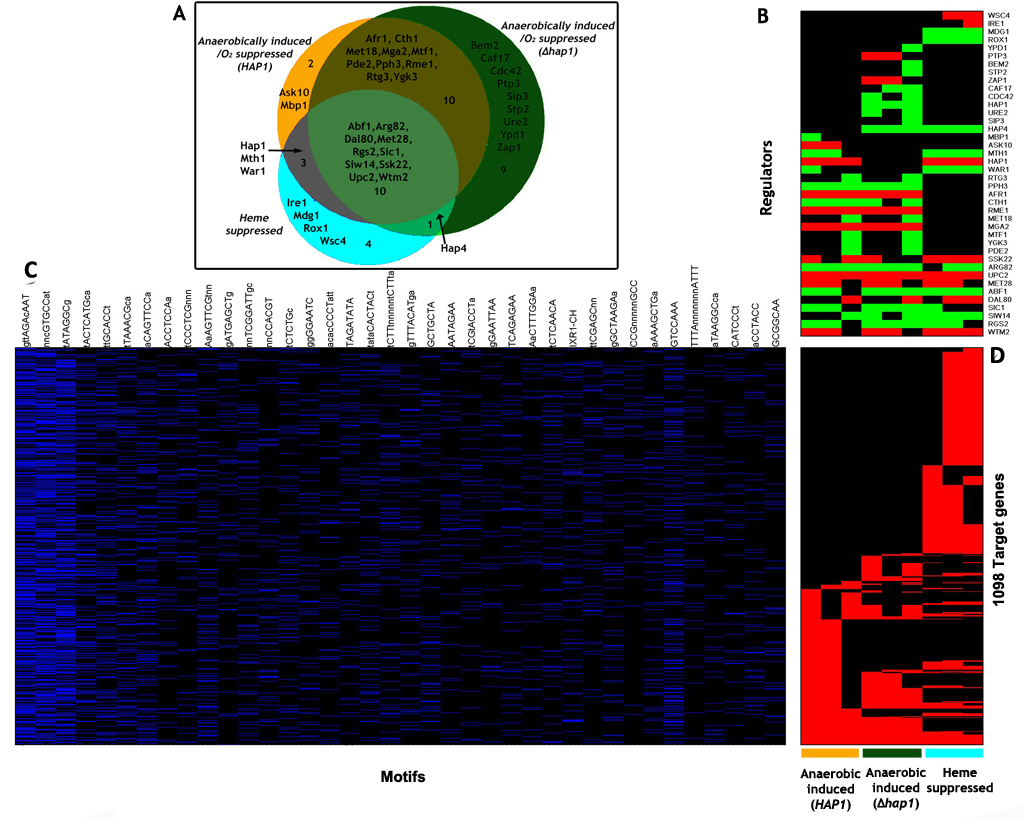


**Figure S4.** Heat map of target genes that are anaerobically induced in *HAP1* and *Δhap1* cells, and those that are induced by Cobalt ion. Significant predictive regulators and sequence motifs are also shown.


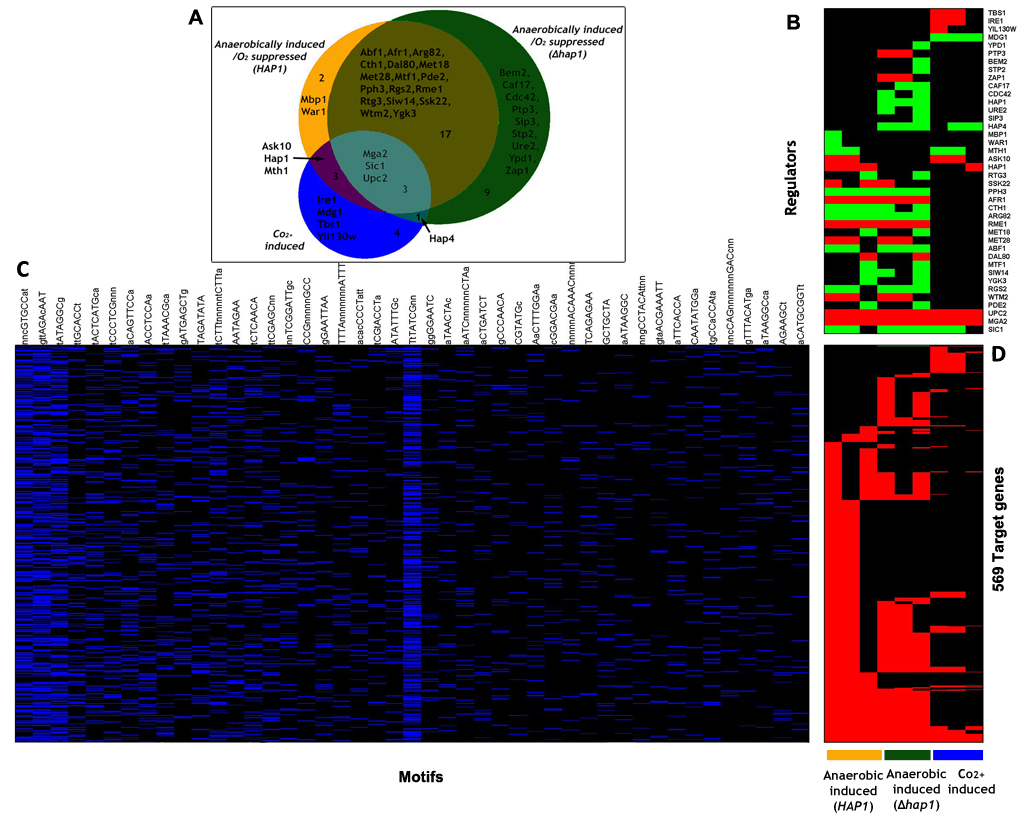


**Figure S5.** Heat map of target genes that are anaerobically suppressed in *HAP1* and *Δhap1* cells, and those that are suppressed by Cobalt ion. Significant predictive regulators and sequence motifs are also shown.


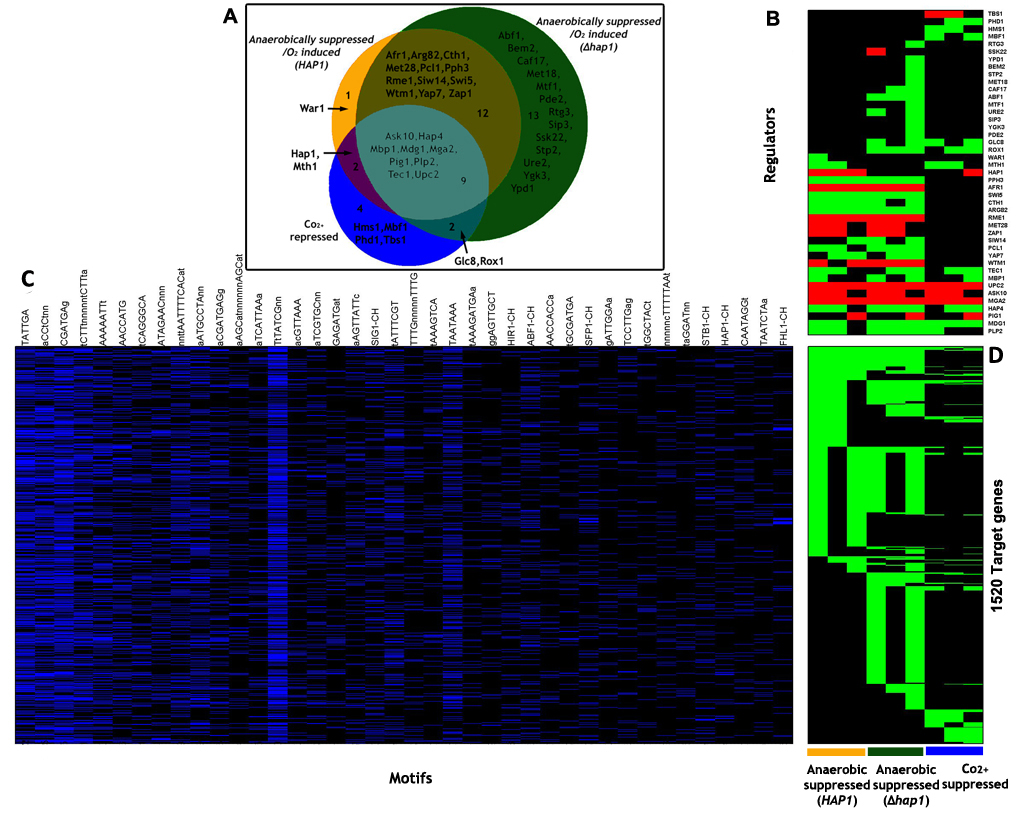


**Figure S6.** Conditions used in microarray expression experiments and identified target genes.


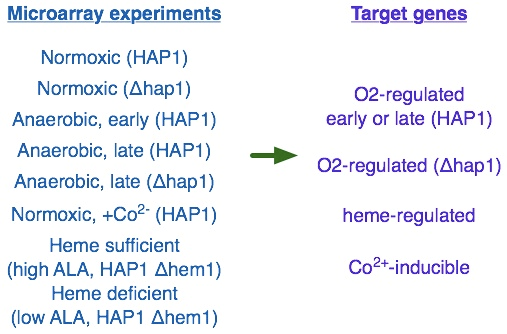


**Figure S7.** Venn diagrams showing the numbers of oxygen-regulated, heme-regulated, and Co^2+^-inducible genes in *HAP1* and ∆*hap1* cells. (A) A Venn diagram illustrating the numbers of hypoxically suppressed (oxygen-induced) genes in *HAP1* and ∆*hap1* cells, and heme-induced genes. (B) A Venn diagram illustrating the numbers of hypoxically induced (oxygen-suppressed) genes in *HAP1* and ∆*hap1* cells, and heme-suppressed genes. (C) A Venn diagram illustrating the numbers of hypoxically induced (oxygen-suppressed) genes in *HAP1* cells at 1.5 or 6 hours after shifting to anaerobic growth conditions. (D) A Venn diagram illustrating the numbers of hypoxically suppressed (oxygen-induced) genes in *HAP1* cells at 1.5 or 6 hours after shifting to anaerobic growth conditions. (E) A Venn diagram illustrating the numbers of hypoxically induced (oxygen-suppressed) genes in *HAP1* and ∆*hap1* cells, and Co^2+^-inducible genes.


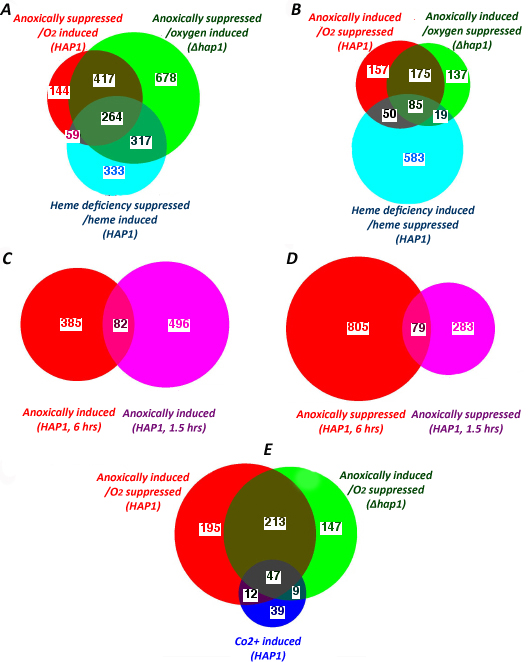


**Figure S8.** The functional categorization of identified oxygen-regulated, heme-regulated, and Co^2+^-inducible genes. This figure illustrates the enriched Gene Ontology process annotations for selected sets of the differentially expressed target gene Venn diagrams of Figure S7. Each row represents a set of genes and each column represents a GO process annotation. The set names are to the left of the color matrix. The GO annotations are shown below the color matrix. Each element of the color matrix illustrates the p-value of enrichment. The colorbar on the right shows the colors used for the range of p-values. The following naming convention is used for the gene sets. Each set represents the intersection of sets of genes that are differentially expressed in different experimental contexts. Each experimental context is represented by an identifier (e.g. Anaerobic represents the hypoxia condition) followed by up or down arrows denoting up-regulation or down-regulation of the genes in that context. The symbol “~” is the logical operations “NOT”, while the up and down arrows specify whether the set of genes up or downregulated in that condition. For example, Anaerobic↑Anaerobic (Δ*hap1*)↑ ~Co↑ refers to the set of target genes that are hypoxically induced both in HAP1 and *∆hap1* cells but are not Co^2+^-inducible. The p-values shown are not corrected for multiple
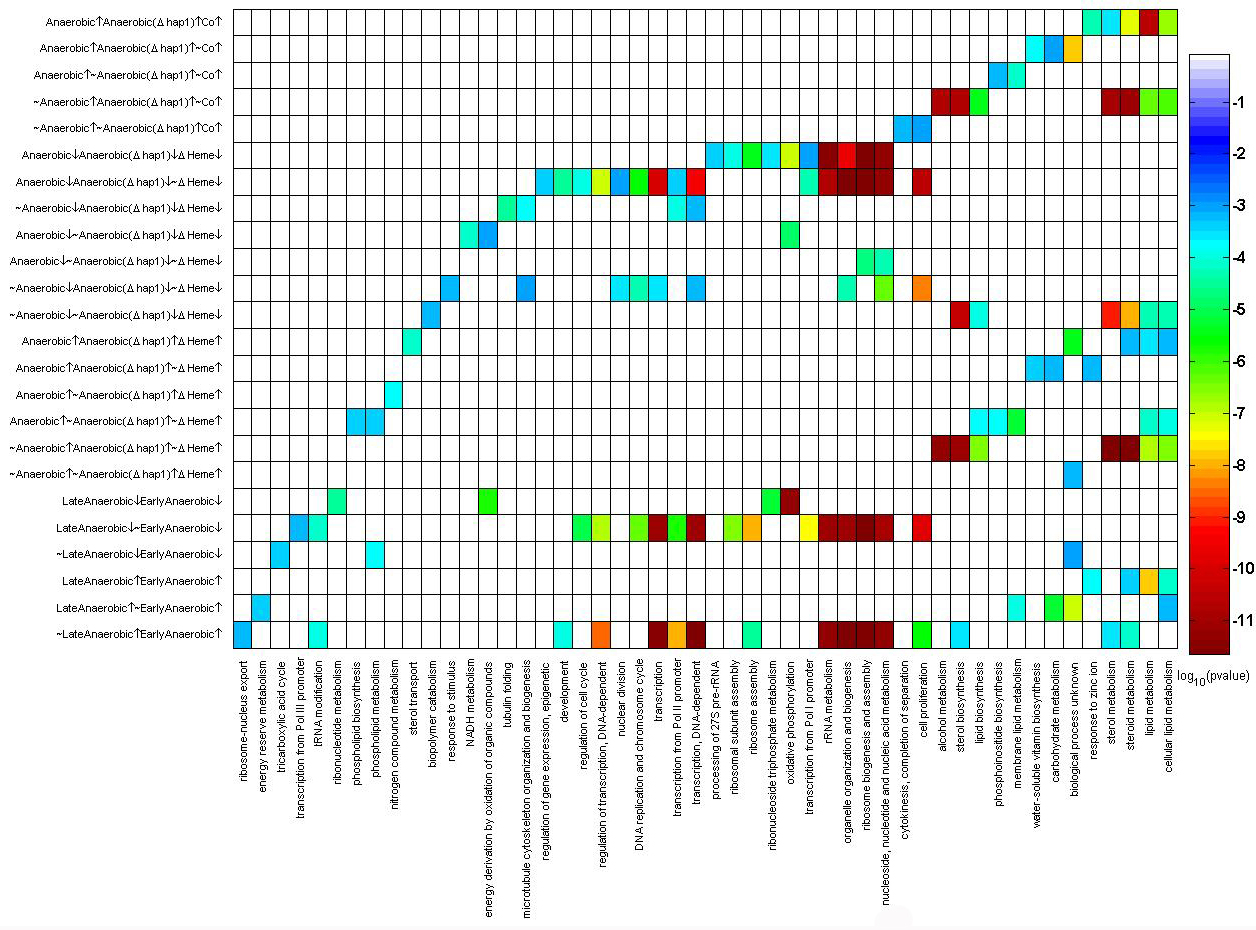
comparisons.

**Figure S9.** Comparison of MacIsaac *et al.* [1] PSSMs to PSSMs learned by MEDUSA. MacIsaac *et al.* [1] used ChIP-chip data to identify potential binding sites for 124 transcription factors. We use the symmetrized Kullback-Leibler (KL) distance to identify the best matching MEDUSA PSSM to each of these 124 PSSMs. The transcription factors are listed in ascending order by the KL distance to the best match. Column 3 shows the MacIsaac *et al.* PSSMs and Column 4 shows the best matching MEDUSA PSSM.


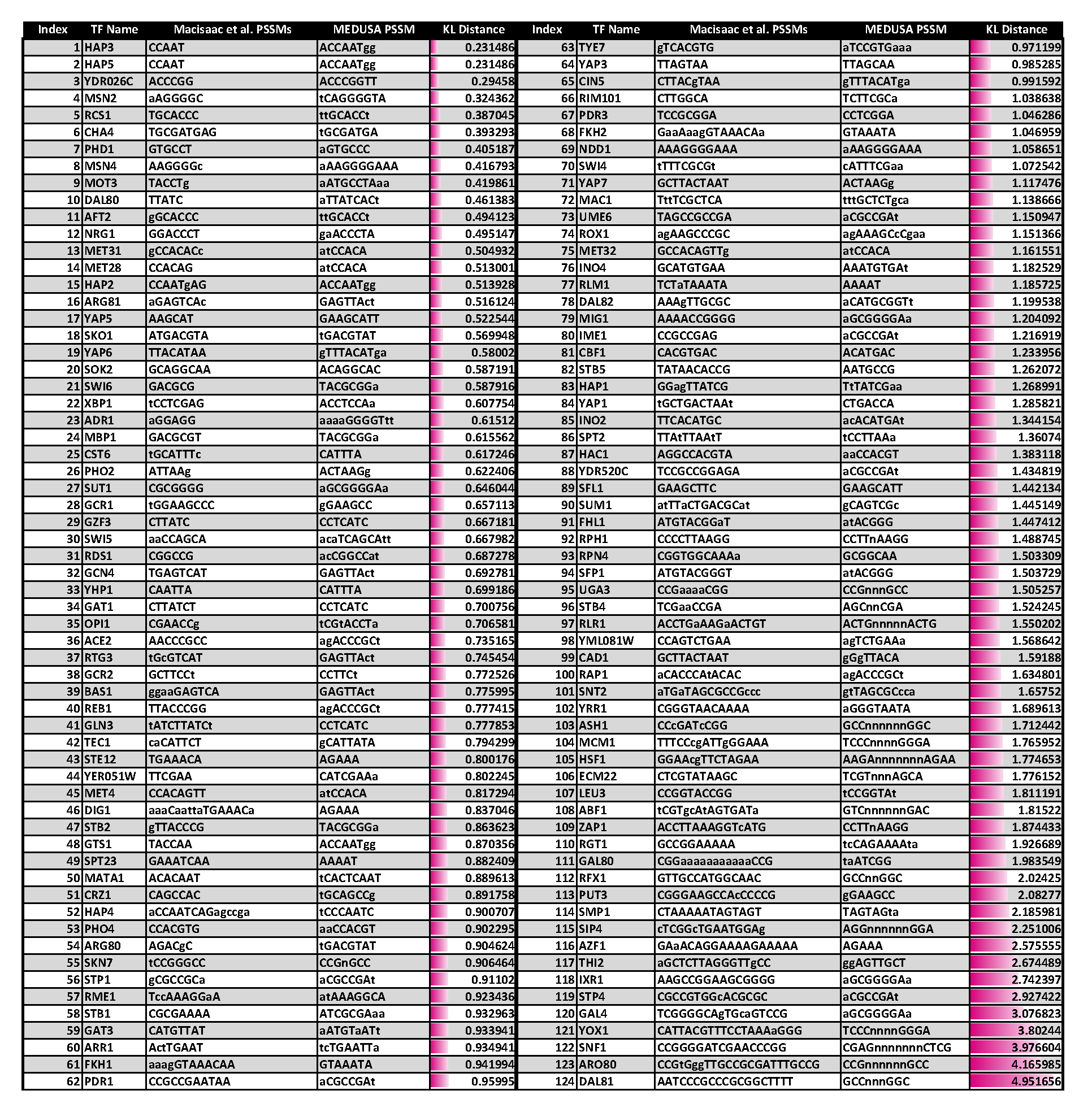


*False discovery rate (FDR) for margin scores*

By applying margin-based scoring to the full list of 506 potential regulators for the up- and downregulated target genes in each experimental condition, we identified 54 regulators (margin score > 0) in the oxygen regulatory network. We use the same sets of up-regulated and down-regulated genes in each experimental condition to estimate the false discovery rates (FDR) for positive margin scores. For each gene set, we randomize the labels of all examples in the gene set based on the class bias of the dataset. We then recalculate the margin score for each regulator. We repeat this procedure 100 times.

**Figure S10.** Positive tail of the empirical null distribution for normalized margin scores. The figure shows the normalized histogram for positive margin scores (margin score > 0) for all gene sets in all the randomization trials. We normalize by dividing the frequency in each bin by the total number of data points (|margin scores > 0| + |margin scores ≤ 0|). The highest observed margin score computed from the randomized data was 0.53. The red points are the 54 regulators with positive margin score using the true labels; if a regulator was identified for multiple gene sets, its most significant *p*-value is shown. We see that most of these points lie far into the positive tail of the distribution.


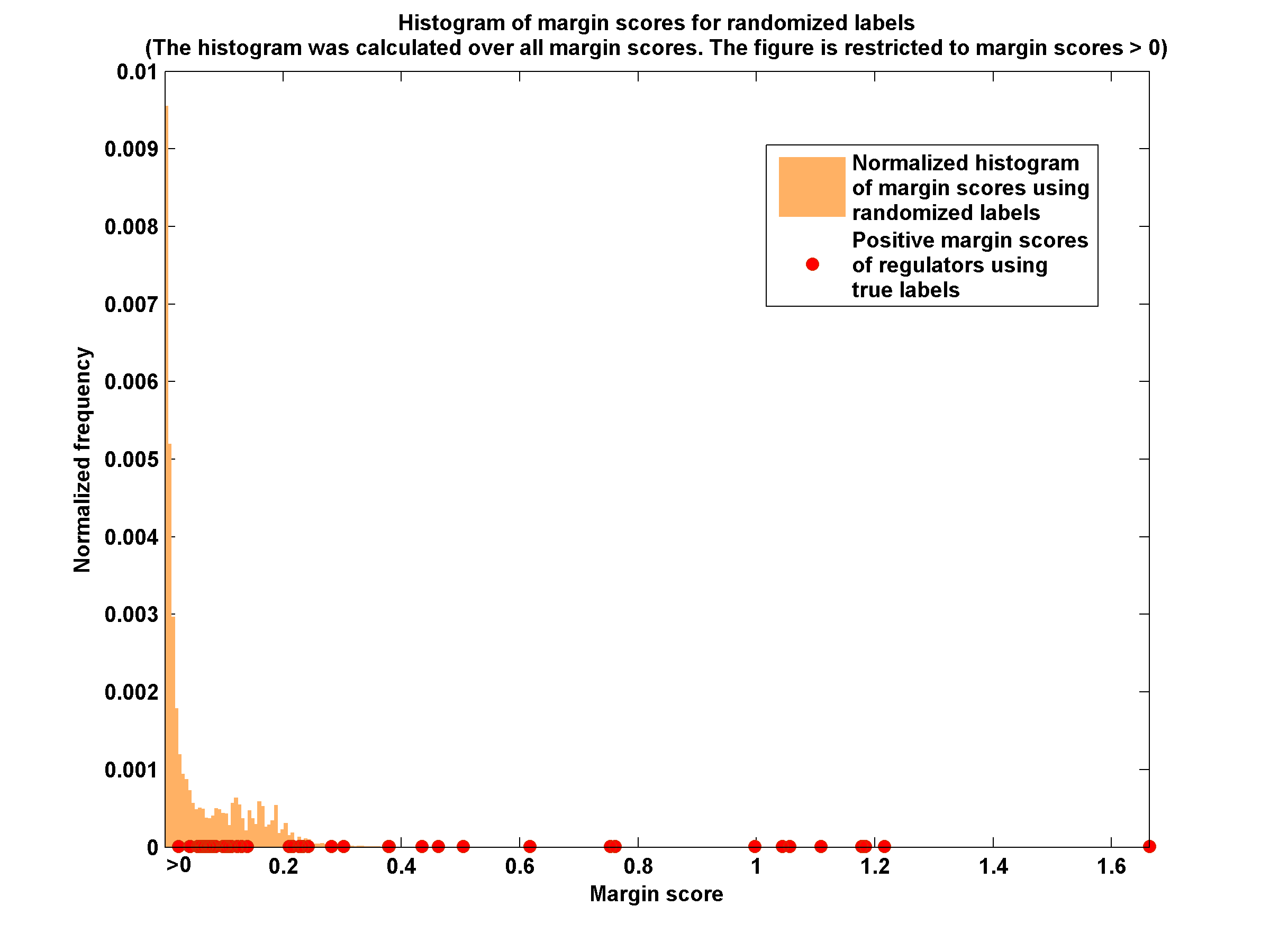


**Figure S11.** Empirical *p*-values for the (normalized) margin score. We calculate a *p*-value for each margin score θ as the fraction of data points in the randomization trials with margin score > θ. The 54 regulators with positive margin scores using true labels have low *p*-values. If a regulator was identified for multiple gene sets, its most significant *p-*value is shown.

**
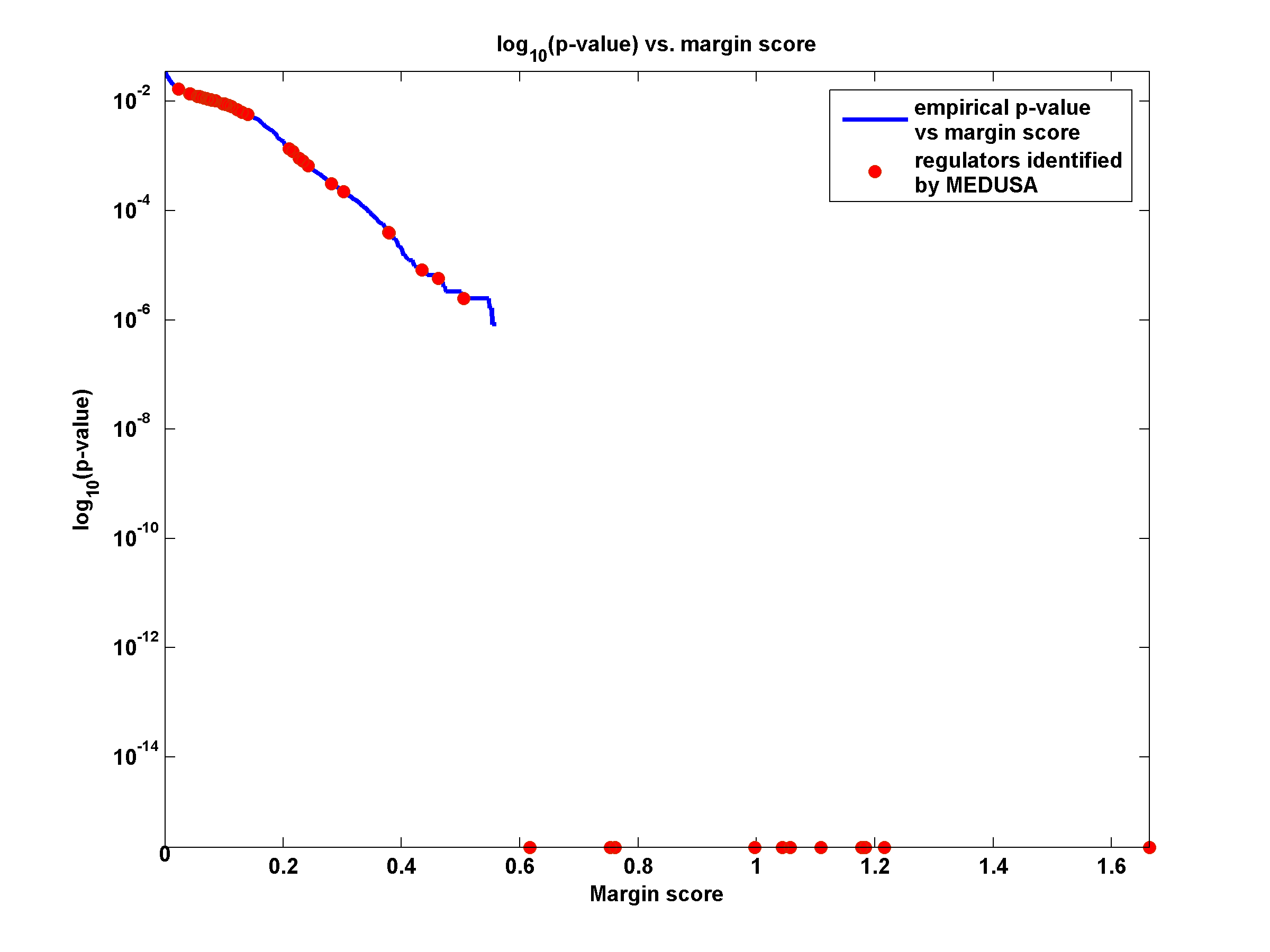
**

**Figure S12.** Estimated false discovery rate for choices of *p*-value threshold for 54 identified regulators. We obtain *p*-values for all 507 regulators in each of the 12 gene sets. We then apply the step-wise Benjamini-Hochberg procedure [2] to this set of *p*-values to obtain the FDR corresponding to each *p*-value cutoff. The figure shows the best margin score, corresponding *p*-value and FDR for the 54 regulators with positive margin score in at least one gene set. The top 16 regulators correspond to very small FDRs and include many known hypoxia regulators such as Upc2, Hap4, Mga2, Rox1 and Hap1, as well as novel regulators.

| **Index** | **Regulator** | **Margin score** | **p-value** | **FDR** |  | **Index** | **Regulator** | **Margin score** | **p-value** | **FDR** |
| --- | --- | --- | --- | --- | --- | --- | --- | --- | --- | --- |
| 1 | NRG1 | 1.6636 | 0 | 0 |  | 28 | CDC42 | 0.1084 | 0.008374 | 0.03701 |
| 2 | RME1 | 1.2162 | 0 | 0 |  | 29 | CAF17 | 0.1026 | 0.008785 | 0.03701 |
| 3 | HAP4 | 1.1839 | 0 | 0 |  | 30 | SSK22 | 0.1008 | 0.008944 | 0.03701 |
| 4 | ABF1 | 1.1778 | 0 | 0 |  | 31 | ASK10 | 0.0982 | 0.009083 | 0.03701 |
| 5 | TBS1 | 1.1089 | 0 | 0 |  | 32 | PIG1 | 0.0855 | 0.010242 | 0.03701 |
| 6 | UPC2 | 1.0565 | 0 | 0 |  | 33 | RGS2 | 0.0796 | 0.010656 | 0.03701 |
| 7 | SWI5 | 1.0436 | 0 | 0 |  | 34 | CTH1 | 0.0781 | 0.010736 | 0.03701 |
| 8 | MET28 | 0.9972 | 0 | 0 |  | 35 | URE2 | 0.077 | 0.010791 | 0.03701 |
| 9 | ROX1 | 0.7607 | 0 | 0 |  | 36 | PLP2 | 0.0726 | 0.011124 | 0.03701 |
| 10 | MBF1 | 0.7527 | 0 | 0 |  | 37 | WTM1 | 0.071 | 0.011236 | 0.03701 |
| 11 | MGA2 | 0.6167 | 0 | 0 |  | 38 | PTP3 | 0.0665 | 0.011622 | 0.03701 |
| 12 | AFR1 | 0.5048 | 2.47E-06 | 0.00091 |  | 39 | WSC4 | 0.0657 | 0.011677 | 0.03701 |
| 13 | DAL80 | 0.462 | 5.75E-06 | 0.00189 |  | 40 | SIC1 | 0.061 | 0.012025 | 0.03701 |
| 14 | ARG82 | 0.4347 | 8.22E-06 | 0.00263 |  | 41 | TEC1 | 0.0609 | 0.01203 | 0.03701 |
| 15 | MTH1 | 0.3794 | 3.94E-05 | 0.01167 |  | 42 | PCL1 | 0.0581 | 0.012313 | 0.03701 |
| 16 | HAP1 | 0.3783 | 4.03E-05 | 0.01167 |  | 43 | FUS3 | 0.0557 | 0.012493 | 0.03701 |
| 17 | MDG1 | 0.3026 | 0.00022 | 0.03701 |  | 44 | YAP7 | 0.0431 | 0.013765 | 0.03701 |
| 18 | WTM2 | 0.2818 | 0.00031 | 0.03701 |  | 45 | WAR1 | 0.0424 | 0.013858 | 0.03701 |
| 19 | HMS1 | 0.2427 | 0.00067 | 0.03701 |  | 46 | RTG3 | 0.0237 | 0.016957 | 0.03701 |
| 20 | SIW14 | 0.2336 | 0.00081 | 0.03701 |  | 47 | YPD1 | 0.0237 | 0.016957 | 0.03701 |
| 21 | ZAP1 | 0.227 | 0.00091 | 0.03701 |  | 48 | BEM2 | 0.0237 | 0.016957 | 0.03701 |
| 22 | GLC8 | 0.2164 | 0.00121 | 0.03701 |  | 49 | STP2 | 0.0237 | 0.016957 | 0.03701 |
| 23 | PHD1 | 0.2098 | 0.00137 | 0.03701 |  | 50 | MET18 | 0.0237 | 0.016957 | 0.03701 |
| 24 | PPH3 | 0.14 | 0.00584 | 0.03701 |  | 51 | MTF1 | 0.0237 | 0.016957 | 0.03701 |
| 25 | IRE1 | 0.1302 | 0.00636 | 0.03701 |  | 52 | SIP3 | 0.0237 | 0.016957 | 0.03701 |
| 26 | MBP1 | 0.1225 | 0.00705 | 0.03701 |  | 53 | YGK3 | 0.0237 | 0.016957 | 0.03701 |
| 27 | YIL130W | 0.1125 | 0.00815 | 0.03701 |  | 54 | PDE2 | 0.0237 | 0.016957 | 0.03701 |

**REFERENCES**

1. MacIsaac KD, Wang T, Gordon DB, Gifford DK, Stormo GD, et al. (2006) An improved map of conserved regulatory sites for Saccharomyces cerevisiae. BMC Bioinformatics 7: 113.

2. Benjamini Y, Hochberg Y (1995) Controlling the False Discovery Rate: A Practical and Powerful Approach to Multiple Testing. Journal of the Royal Statistical Society Series B (Methodological) 57: 289-300.
